# Efficacy of Low Dose of Dihydroquercetin (DHQ) in Two Genetic Models of Neurodegeneration: Insights from FUS[1-359]-Tg and APPswe/PS1dE9 Paradigms

**DOI:** 10.3390/cells15171598

**Published:** 2026-09-02

**Authors:** Ekaterina Lysikova, Kseniia Sitdikova, Aigerim Makhambetova, Kirill Chaprov, Anna Gorlova, Andrey Kostin, Polina Novikova, Alexei Lyundup, Sholpan Askarova, Michail S. Kukharsky, Alexey Deykin, Tatyana Strekalova

**Affiliations:** 1 Laboratory of Medical Sciences, Medical Research Council, London W12 0HS, UK; k.lysikova@lms.mrc.ac.uk; 2 Center for Life Sciences, National Laboratory Astana, Nazarbayev University, Astana 010000, Kazakhstan; sitdikova.kk@gmail.com (K.S.); aigerim.makhambetova@nu.edu.kz (A.M.); shaskarova@nu.edu.kz (S.A.); 3 Laboratory of Genetic Technology and Gene Editing for Biomedicine and Veterinary, National Research Belgorod State University, 308015 Belgorod, Russia; alexei@deikin.ru; 4 Institute of Physiologically Active Compounds at Federal Research Center of Problems of Chemical Physics and Medicinal Chemistry of Russian Academy of Sciences, 142432 Chernogolovka, Russia; chaprov@ipac.ac.ru (K.C.); kukharskym@ipac.ac.ru (M.S.K.); 5 Research and Education Resource Center, Peoples Friendship University of Russia (RUDN University), 117198 Moscow, Russia; gorlova-av@rudn.ru (A.G.); kostin-aa@rudn.ru (A.K.); liundup-av@rudn.ru (A.L.); 6 Department of Normal Physiology, Sechenov University, 117198 Moscow, Russia; novikova_p_a1@student.sechenov.ru; 7 Department of Psychiatry and Neuropsychology, Maastricht University, Universiteitsinsel 50, 6229 ER Maastricht, The Netherlands

**Keywords:** Alzheimer’s disease (AD), amyotrophic lateral sclerosis (ALS), dihydroquercetin, antioxidants, APPswe/PS1dE9 mice, FUS[1-359]-Tg mice

## Abstract

Dihydroquercetin (DHQ), a powerful antioxidant and regulator of cellular metabolism, was proposed for therapy of neurodegenerative disorders. Alzheimer’s disease (AD) and amyotrophic lateral sclerosis (ALS) are serious neurodegenerative disorders with oxidative stress as an overlapping feature and unmet therapeutic needs. To date, few studies have explored the efficacy of DHQ in animal models of genetically driven neurodegeneration. Here, APPswe/PS1dE9 (APP/PS1) mice and their wild-type (WT) littermates were orally administered DHQ (0.6 mg/kg/day) for four months, starting at eight months of age. At the age of 12 months, behavioral evaluation was performed, followed by brain staining with Congo red for amyloid plaque scoring and immunohistochemical analysis of GFAP-positive cells for the assessment of astrogliosis. Malondialdehyde (MDA) levels in the prefrontal cortex were studied as a marker of oxidative stress. Second, two-month-old FUS[1-359]-Tg mice, which recapitulate the hallmarks of ALS, received DHQ for 1.5 months and were investigated for general physiological parameters, the onset of paralysis, motor functions, and density of motor neurons in the spinal cord. DHQ-treated APPswe/PS1dE9 mutants displayed a decrease in amyloid plaque density of small size (≤100 μm) in the cortex and thalamus, had normalized MDA levels, improved conditioned taste aversion and Y-maze learning, and ameliorated anxiety measures, whereas their hippocampus-dependent step-down and pellet displacement performance remained impaired. In the second study, DHQ-treated FUS[1-359]-Tg mice showed rescued density of spinal cord neurons, normalized liquid and diet intake, and improved coat state, while the onset of paralysis and motor scores were not significantly ameliorated. Thus, chronic administration of low doses of DHQ exerted neuroprotective effects in both AD and ALS genetic models, which partially translated to reduced manifestations of these diseases.

## 1. Introduction

The use of antioxidants, particularly flavonoids, is considered a promising strategy for preventing and treating neurodegenerative disorders [1,2]. Their effects are thought to occur via direct action on oxidative stress and inflammatory processes contributing to neurodegeneration [2,3,4]. In particular, it is well established that elevated levels of reactive oxygen species (ROS) in the CNS cause damage to DNA, proteins, and lipids, resulting in neuronal death that drives heterogeneous neurodegenerative conditions, such as Alzheimer’s disease (AD), amyotrophic lateral sclerosis (ALS), and others [2,3,4]. Dihydroquercetin (DHQ), also known as taxifolin, a 3,5,7,3,4-pentahydroxy flavanone (Supplementary Figure S1), belongs to the flavonoid family and was discovered as a naturally derived compound with high antioxidant activity against ROS and nitrosative stress [5,6]. The extraordinary antioxidant properties of DHQ account for the outstanding vitality of larch species, such as *Larix sibirica* (Siberian larch). Its broad availability in common plants, vegetables, onions, and citrus fruits and its availability from plant waste position this compound as an attractive target for affordable and effective medicinal use [1,7,8]. Since its discovery, a wide range of pharmacological activities of DHQ have been reported to be valuable for therapeutic purposes, including anti-inflammatory, hepatoprotective, antioxidant, antiangiogenic, antidiabetic, cardioprotective, and neuroprotective properties [8,9]. However, the practical use of DHQ may be greatly limited by its low water solubility, limited bioavailability, and rapid degradation, which have been attempted to be overcome in many studies [10,11].

Previous studies have suggested that the neuroprotective properties of DHQ are associated with its flavonoid structure, particularly its hydroxylation profile, metal-chelating activity, anti-amyloid effects, and related physicochemical properties [8]. For example, consumption of 3% DHQ chow for 13 months by Tg-SwDI mice, characterized by amyloid β (Aβ) deposition in the cerebrovasculature, inhibited the intracerebral production of Aβ through suppressing the ApoE–ERK1/2–amyloid-β precursor protein axis; lowered gene expression of pro-inflammatory cytokines, tumor necrosis factor (TNF), interleukins IL-6 and IL-1β, and oxidative stress-responsive genes CuZn-SOD and Mn-SOD; and reduced microglia activation and glutamate levels in the hippocampus and cortex [9]. In a rat scopolamine model of dementia, pretreatment with DHQ at a dose of 5 mg/kg/day mitigated the scopolamine-induced increase in acetylcholinesterase activity; counteracted increased myeloperoxidase activity, neuronal death, and oxidative stress markers of augmented lipid peroxidation and nitrite; and rescued the activity of superoxide dismutase, catalase, and glutathione [12]. In an aluminum chloride (AlCl_3_)-induced neurotoxicity model of AD, two-week intraperitoneal concomitant dosing of rats with DHQ (1, 2, and 5 mg/kg/day) attenuated memory impairment in the Morris water maze and changes in motor and emotionality scores, as well as normalized levels of malondialdehyde (MDA), nitrite, glutathione, the activity of myeloperoxidase, and the expression of a key mediator of innate immune and neuroinflammatory signaling, Toll-like receptor 4 (TLR4) [13].

The mechanisms underlying the neuroprotective effects of DHQ have also been demonstrated in vitro. The ability of DHQ to suppress amyloid β production was further demonstrated in the mouse neuroblastoma N2aSwe cell line, which expresses the human amyloid precursor protein (APP) with the Swedish mutation. This was accompanied by an elevation in both the expression and enzymatic activity of sirtuin 1 (SIRT1), a neuroprotective NAD^+^-dependent deacetylase that regulates oxidative stress and mitochondrial function, and a decrease in β-site APP-cleaving enzyme 1 (BACE1) protein expression, which is an important factor in AD [14]. The beneficial effects of DHQ have been demonstrated in a cellular model of AlCl3-induced toxicity [13]. In addition, in an in vitro model of ischemia, pre-incubation with DHQ decreased the apoptosis of mouse cerebral cortical cells and reduced the expression of inflammation-related genes, such as interleukins IL-1β, IL-6, IL-10, nuclear factor kappa B (NF-κB), as well as apoptosis-related genes, including caspase-3 (Casp-3) and B-cell lymphoma 2 (Bcl-2) [15].

Importantly, while many in vivo effects of DHQ were attributed to a dose range of 5–10 mg/kg/day [12,16], several studies have revealed physiological activities of chronic DHQ administration at much lower doses in the range of 0.25–1 mg/kg/day, thus opening the possibility of employing this compound as an aqueous solution as the water solubility threshold [17]. For instance, in a rat rotenone-induced model of Parkinson’s disease, ten-day oral post-treatment with 0.25, 0.5, and 1.0 mg/kg of DHQ attenuated dysfunction of key markers involved in mitochondrial bioenergetics, dopamine metabolism, oxidative stress, glutamate homeostasis, and neuronal survival, including mitochondrial complex I, lactate dehydrogenase, tyrosine hydroxylase, monoamine oxidase, glutamine synthetase, and Na^+^/K^+^-ATPase in the hippocampus and cortex of Wistar rats [18].

In a study with a similar design, low doses of DHQ suppressed the rotenone-induced upregulation of pro-inflammatory cytokine IL-1β, NF-κB, and inhibitor of kappa B (IκKB) expression in the striatum and reduced parkinsonian symptoms, such as bradykinesia, catalepsy, postural instability, impaired locomotor behavior, and tremor. In this model, short oral administration of DHQ at a dose of 0.5 mg/kg restored dopamine levels and monoamine oxidase activity in the hippocampus and cortex of rats after rotenone-induced neurotoxicity [19]. In addition, in a rat model of ischemia, intravenously administered DHQ at doses of 0.1 and 1 mg/kg inhibited brain leukocyte infiltration and inflammation by decreasing the central components of the ischemia-induced neuroinflammatory cascade: cyclooxygenase-2 (COX-2), inducible nitric oxide synthetase (iNOS), and myeloperoxidase (MPO) activities, and by antagonizing NF-kB activation [20]. Finally, a clinical study demonstrated the anti-inflammatory effects of orally administered DHQ at a dose of 1 mg/kg/day [21].

Based on the available literature, we hypothesized that low doses of chronically administered DHQ might exert a neuroprotective effect in genetic models of neurodegeneration. It should be noted that the currently available evidence of the biological effects of DHQ in neurological conditions is limited by toxicity models. Therefore, we chose to study the potential therapeutic effects of DHQ in two genetic models: APPswe/PS1dE9 mice, a model of AD, and FUS[1-359]-Tg mice, a model of ALS. We sought to probe the efficacy of the DHQ dose that would allow the use of the aqueous solution for dosing animals, given the limitations of other routes of chronic treatment based on lengthy drug administration [22].

Here, a DHQ dose of 0.6 mg/kg/day was selected based on previous studies demonstrating neuroprotective and anti-inflammatory efficacy following oral administration within the range of 0.25–1.0 mg/kg/day [18,19]. An intermediate dose was chosen to maximize the likelihood of achieving therapeutic efficacy while minimizing the risk of adverse effects during prolonged administration, which was particularly important given the chronic treatment protocol and the use of aged and diseased animals.

DHQ was administered continuously via drinking water because the compound reaches peak plasma concentrations rapidly after oral administration and is cleared from circulation within a short time; no marketable tissue accumulation has been reported [23,24]. Continuous voluntary intake by mice, which drink throughout the light–dark cycle, was considered the most appropriate for maintaining sustained systemic exposure. The concentration of DHQ in the drinking water selected for the study was more than two orders of magnitude below its reported aqueous solubility threshold of 1 mg/mL, ensuring its complete dissolution under the employed experimental conditions. Tap water was used as a vehicle, as a compromise between providing molecule stability and the physiological needs of animals that do not allow the use of distilled or deionized water. Although higher doses of DHQ have been employed in some studies, increasing the administered dose was not suggested to proportionally increase brain exposure because of the limited bioavailability of the compound [17,24,25]. We also considered the significant increase in blood brain barrier (BBB) permeability in Alzheimer’s disease [26] and ALS [27,28], suggesting that considerable augmentation of the actual DHQ concentrations in the brain tissue of the dosed APPswe/PS1dE9 mice and FUS[1-359]-Tg mice orally dosed with DHQ can be anticipated compared to previously reported values measured in healthy animals.

First, we employed APPswe/PS1dE9 mice, which are widely used to model AD pathology [29]. APPswe/PS1dE9 mice express a mutant human amyloid precursor protein (APP) gene with a Swedish mutation (K594N/M595L) and a mutant human presenilin1 gene (PS1) with deleted exon 9 (PS-1dE9) and develop extracellular amyloid plaques in the brain, peaking at the age of 10 months [29,30]. These transgenic mice also recapitulate key AD-like abnormalities in hippocampus-dependent performance, elevated anxiety, microglial activation, overexpression of IL-6, TNF, and IL-1β, and accumulation of MDA, an oxidative stress marker [31,32,33]. Various treatments with antioxidants and anti-inflammatory properties have recently been shown to counteract the development of these hallmarks of AD, such as stem cell therapy [31,32] and antioxidants such as fullerene C60 and fullerenol C60(OH)_24_) [33]. Prolonged administration of either antioxidant treatment in the latter study improved cognitive and emotional behaviors, decreased amyloid plaque density, and diminished astrogliosis [33].

Here, APPswe/PS1dE9 (APP/PS1) mice and their wild-type (WT) littermates were orally administered DHQ at a selected dose dissolved in regular water for four months, starting at eight months of age. At the age of 12 months, behavioral assessment was performed using the spatial form of the Y-maze, conditioned taste aversion model, pellet displacement test, step-down avoidance test, and classic tests for anxiety and locomotion, as described elsewhere [31,32,33]. This was followed by brain staining with Congo red for amyloid plaque scoring and immunohistochemical analysis of GFAP-positive cells for assessment of astrogliosis; malondealdehyde (MDA) levels in the prefrontal cortex were studied as a marker of oxidative stress, according to previously established parameters in 12-month-old APPswe/PS1dE9 mice [31,32,33,34].

Next, we used FUS[1-359]-Tg mice, a genetic model of ALS in which the expression of human truncated fused in sarcoma (FUS) protein in neuronal cells recapitulates key symptoms of ALS [35,36,37]. This model recapitulates the physiological consequences of human mutations in FUS, an RNA-binding protein that is a frequent cause of ALS [38]. In the FUS[1-359] transgenic model, disease pathogenesis was attributed primarily to synaptic dysfunction and pathological protein aggregation [39], compromised cellular stress responses, reduced expression of ion channels and transporters required for synaptic function, and impaired neuronal activity [40]. FUS[1-359]-Tg mice display disrupted function of NEAT1-dependent paraspeckles, altered microRNA biogenesis, increased oxidative stress, mitochondrial dysfunction, and apoptosis [35,41,42].

Many of these pathogenic mechanisms converge on oxidative stress and neuroinflammation, as NEAT1 promotes IL-1β and TNF production, pyroptosis, and macrophage activation [43,44]. FUS[1-359]-Tg mice showed activated microglia and NLRP3 inflammasome, leading to caspase-1- and IL-1β-mediated motor neuron injury [45,46,47]. Notably, DHQ suppresses TLR4/NF-κB and NLRP3/caspase-1 signaling, inhibits microglial pyroptosis, and activates the Nrf2/HO-1 antioxidant pathway [9,48,49,50,51,52], providing a rationale for evaluating this compound in the FUS[1-359]-Tg model. Previous studies have demonstrated the partial efficacy of treatments with anti-inflammatory and antioxidant properties in slowing disease progression in FUS[1-359]-Tg mice, including resveratrol [38], thiamine compounds [36,37], and stem cell therapy [31,32]. In the present study, two-month-old FUS[1-359]-Tg mice received DHQ via drinking water for 1.5 months, and the onset of paralysis, general physiological parameters and coat state, as well as motor functions and density of motor neurons in the spinal cord were studied.

We found that chronic administration of a low dose of DHQ dissolved in water exerted partial ameliorative effects in both AD and ALS genetic models, suggesting potential rationale for further studies on its potential therapeutic usefulness in these pathologies.

## 2. Materials and Methods

### 2.1. Animals

APPswe/PS1dE9 (APP/PS1) female mice and their wild-type (WT) littermates bred on a C57Bl6J genetic background were 8 months old at the start of the experiment (see below). Upon transport from FDA-certified Bioresource Collection of Centre for Collective Use (FFSG-2024-0020) IPAC RAS (http://www.ipac.ac.ru/index.html, accessed on 25 August 2026), the animals were housed in groups of three mice per cage (30 × 20 × 15 cm) under standard laboratory conditions, as described elsewhere [33]. Female transgenic animals were selected as they exhibit greater vulnerability to AD pathology than males [29,30], which is consistent with epidemiological data showing a higher lifetime risk of developing AD [2]. FUS[1-359]-Tg male mice and their wild-type (WT) littermates bred on the C57BL/6J genetic background were 2 months old at the start of the study (see below) and were provided by FDA-certified Bioresource Collection of Centre for Collective Use (FFSG-2024-0020) IPAC RAS and single-housed (size of a cage 30 × 20 × 15 cm) under standard laboratory conditions, as previously described [37]. In both studies, the mice were allowed to acclimatize for seven days prior to the start of the experiment. In both studies, the standard laboratory conditions implied 22 ± 1 C° room temperature, 55 ± 2% humidity, food and water ad libitum, light intensity during the light phase in average 150 Lx, 12 h: 12 h lighting conditions. Animals were housed under a reverse light/dark cycle, with lights on at 20:00 and off at 8:00, and all behavioral experiments were conducted during the dark (active) phase of the day. For environmental enrichment, two face tissues were placed per cage so that animals could build their nests; further enrichment was not used to preclude potential interference with the behavioral analysis.

Potential confounding factors in the behavioral analysis were controlled as described previously [3]. All animal procedures were carried out in accordance with the European Guidelines for the use of animals in research (2010/63/EU) and the Registration of an Institutional Review Board (IRB) of the Center for Life Sciences of Nazarbayev University, following approvals from the Local Ethics Committee PE “National Laboratory of Astana”, Nazarbayev University, dated 20 March 2023, N02-2023 and 21 November 2023, N05-2023, concerning animal care and welfare, and in line with ARRIVE guidelines (accessed the last time on 1 March 2025). All efforts were undertaken to ensure compliance with the above-mentioned regulations concerning human endpoints in animal research that were established before the start of the study.

The animals were closely monitored throughout the study. Therefore, the mice were examined at least twice daily starting at 85 days. Humane endpoints included severe motor dysfunction, that is, including to right within 30 s and inability to reach food or water, or showing severe weight loss (>20%). Animals meeting these criteria were excluded from the experiment, euthanized using an overdose of isoflurane, followed by cervical dislocation, in accordance with institutional ethical approval.

### 2.2. Study Flow

**Figure 1:**
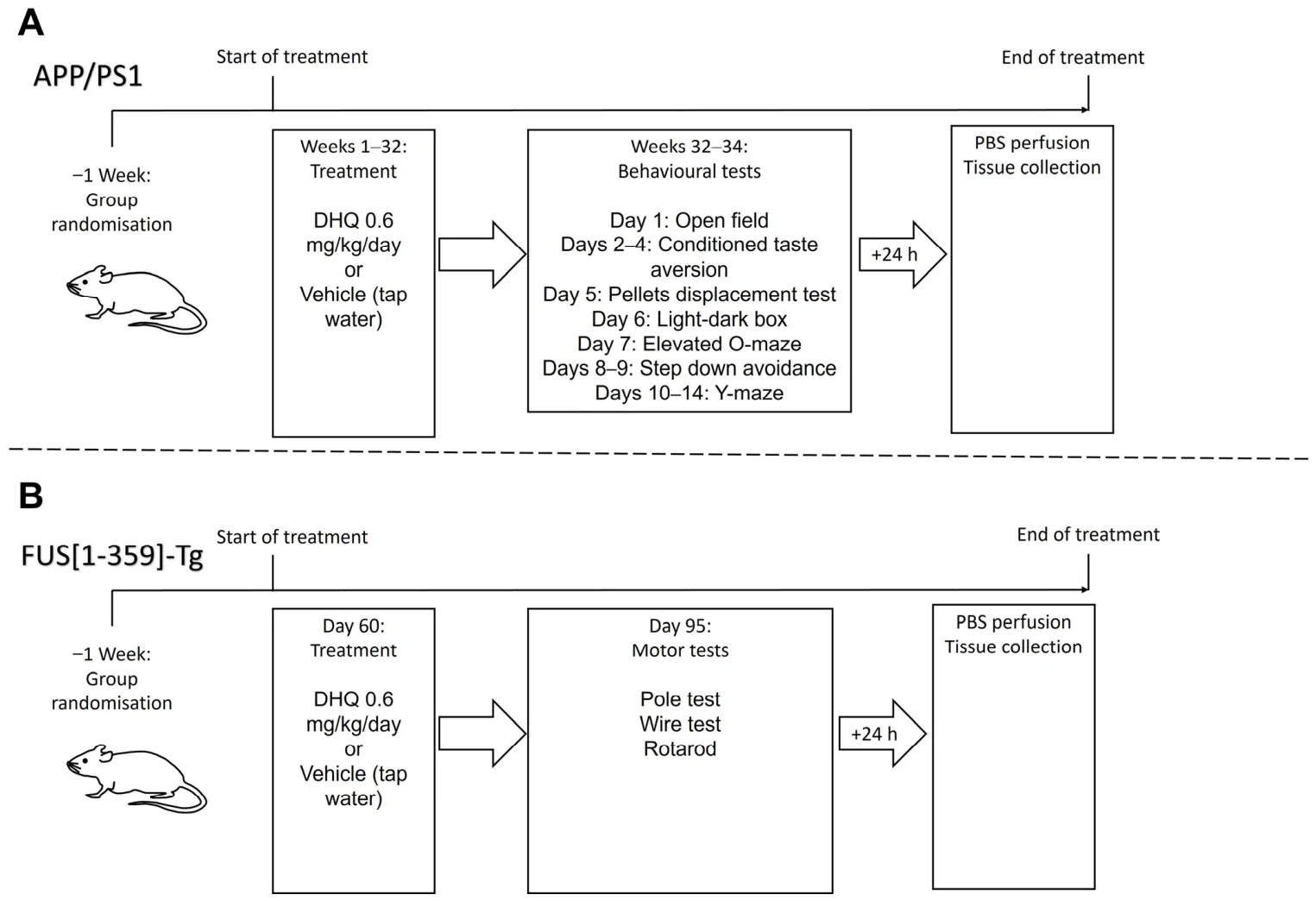
Experimental design. (**A**) APP/PS1 mice and their WT littermates were treated with DHQ or the vehicle from the age of 8 months. At the age of 12 months, APP/PS1 animals were compared to WT littermates in consecutive behavioral tests: open field on day 1, conditioned taste aversion on days 2–4, pellets displacement on day 5, light–dark box on day 6, elevated O-maze on day 7, step-down avoidance on days 8–9 and memory formation in Y-maze on days 10–14. 24 h after the last test, mice were culled, and their brains were collected. (**B**) FUS[1-359]-Tg mice and their WT littermates were divided into the WT–vehicle, FUS[1-359]-Tg–vehicle, and FUS[1-359]-Tg–DHQ groups. Mice received treatment from the age of 60 days, motor function was tested at the age of 95 days in the pole test, wire test, and rotarod, and 24 h later, mice were culled and the lumbar region of the spinal cord was collected. DHQ—dihydroquercetin; PBS—phosphate-buffered saline. The horizontal dashed line separates the two independent experimental cohorts.

APP/PS1 mice and their WT littermates were assigned to groups receiving DHQ dissolved in tap water (DHQ, 0.6 mg/kg/day, *n* = 9 for WT, *n* = 6 for APP/PS1) or regular tap water (*n* = 9 for WT, *n* = 5 for APP/PS1) (Figure 1A). A total of 29 animals were used in the first experiment. Group sizes were determined based on practical and ethical considerations associated with the use of APPswe/PS1dE9 and FUS[1-359]-Tg mice [31,32,33,34,36,37,53,54,55], including the limited availability of age- and sex-matched transgenic animals, breeding constraints, and compliance with the 3Rs principle. Unequal group sizes reflected the genotype distribution obtained from breeding, and all available littermates of the appropriate genotype and age were included rather than excluding animals solely to achieve a balanced group. In both studies, animals were randomized for the upcoming treatment according to their body weight and date of birth.

A priori power analysis was performed. Group sizes were planned based on the effect sizes observed in previous studies employing the same mouse lines and comparable readouts, in which genotype- and treatment-related differences in amyloid plaque density, MDA concentration in the prefrontal cortex, spinal motor neuron counts, motor performance, and hippocampus-dependent behaviors were consistently of large magnitude (Cohen’s f ≥ 0.6) [31,32,33,34,36,37,53,54]. The corresponding power calculations, performed in G*Power 3.1.9.7 with α = 0.05, 1−β = 0.80, and f = 0.6, indicated that the two-way (genotype × treatment) design of the APP/PS1 study required a total of 25 animals (numerator df = 1, four groups; λ = 9.00, critical F_(1,21)_ = 4.32, actual power = 0.816), whereas the one-way design of the FUS[1-359]-Tg study required a total of 30 animals (numerator df = 2, three groups; λ = 10.80, critical F_(2,27)_ = 3.35, actual power = 0.800). The number of animals enrolled was 29 and 40, respectively. The adequacy of the sample sizes was subsequently confirmed by a sensitivity power analysis (see Section 2.12).

Liquid intake was measured on days 1, 2, and 3 of dosing and weeks 1, 2, 4, 8, 12, and 16; no group changes were observed (Supplementary Table S1). After 4 months of supplementation with DHQ, mice were subjected to a series of behavioral tests for two weeks, including the open field, conditioned taste aversion, pellet displacement test, light–dark box, elevated O-maze, step-down avoidance, and Y-maze, with at least 24 h time intervals between tests (Figure 1A). Thereafter, all mice were euthanized, and their brains were collected for histological analysis and MDA measurement (see below).

FUS[1-359]-Tg mice received either DHQ dissolved in tap water (0.6 mg/kg/day, *n* = 14) or normal tap water (*n* = 14) from the age of 2 months (60 days), and WT littermates received tap water (*n* = 12) (Figure 1B). A total of 40 animals were used in this study. At 95 days, water and diet intake were measured, and the coat state was scored (1—poor, 5—excellent) [56]. Liquid intake was measured on days 1, 2, and 3 of dosing and weekly on weeks 1–6, no group changes were observed (Supplementary Table S2). Locomotor functions were assessed using the pole test, wire test, and rotarod model, and the number of mice that developed symptoms (limb paralysis) was compared among the mutant groups. All mice were culled at the age of 3.5 months, their spinal cords were dissected, and the lumbar regions were harvested for histological analysis. Our study was conducted in accordance with internationally accepted guidelines for preclinical ALS studies, including the use of well validated genetic models of ALS, blinded assessment, and a broad range of predefined readouts [57].

At the end of the experiment, the mice were terminally anesthetized with isoflurane and perfused with phosphate-buffered saline (PBS; pH 7.4) solution. The experimenters were blinded to the group assignments in all assays. This was achieved by using the ID of each mouse, in which only individual numbers not containing treatment and genotype were used.

### 2.3. DHQ Preparation and Administration

The experimental solution replaced the normal drinking water. DHQ (Sigma-Aldrich, Saint Louis, MO, USA) was dissolved in tap water and the solution was changed every 4–5 days, liquid intake was monitored as described elsewhere [58,59]. Specifically, the mice were weighed every week, and their individual liquid intake for 24 h was evaluated by weighing the bottles. We used bottles that ensured a minimal spillage of 0.1 mL, which is <5% of the daily liquid intake, as validated in our previous experiments with drinking tests and dosing of mice via drinking water [22]. Several precautions were undertaken to increase the accuracy of dosing, such as filling bottles a few hours prior to use and storing them in the laboratory room upside down, omitting the use of detergent while washing bottles that can be repulsive for mice, and regular evaluation of liquid intake and body weight [22,30,31,32,33,58,59].

The DHQ concentration was adjusted based on the daily liquid intake to achieve a target dose of 0.6 mg/kg/day. The resulting concentration was approximately 0.0045 mg/mL, which was more than two orders of magnitude below its reported aqueous solubility limit of 1 mg/mL [17,60], ensuring complete dissolution and chemical stability under the experimental conditions (approximately 22 °C, tap water had a pH of approximately 7.0).

Tap water was chosen instead of deionized or distilled water as the DHQ solvent because prolonged consumption of deionized/distilled water, which could potentially preserve DHQ stability [60,61,62], may adversely affect fluid and electrolyte homeostasis in rodents during chronic ad libitum administration [63,64]. To minimize potential degradation, DHQ solutions were prepared fresh twice weekly and used at room temperature ≤ 22 °C in specialized drinking bottles without metal components, which reduced metal-catalyzed oxidation and air exchange. As the tap water used for DHQ administration had a neutral pH and contained acceptable levels of dissolved metal ions, its use was considered adequately appropriate to preserve DHQ stability, given the above-indicated physiological constraints. The DHQ solution used in this study was confirmed not to exhibit measurable changes in pH, as evaluated at the start of our study. Liquid intake was monitored by weekly measurements of 24 h liquid consumption, as described elsewhere [22,33]. No significant group differences in liquid consumption were observed, indicating normal drinking behavior in DHQ-treated mice (see Supplementary Materials, Tables S1 and S2).

### 2.4. Behavioral Assays

#### 2.4.1. Open Field

Animals were studied in the open field test using a grey square polyvinyl chloride box (45 × 45 × 45 cm, Technosmart, Rome, Italy) under illumination intensity of 25 lx, as described elsewhere [59,65]. A shadow-free illumination system was used as previously validated to preclude artifacts in the automated scoring of mouse behavior [59,65]. Mice were allowed to acclimatize to the testing room for at least 1 h prior to the experiment. The computer used for scoring was placed in a separate room to prevent the possible effects of sound on mouse behavior. The arena was carefully cleaned between trials. The animals were positioned in the corner of the arena, facing the wall, and monitored for 15 min. The number of crossed sectors (5 × 5 cm each), defined as the entry of all four paws into an adjacent sector, and the duration of freezing, defined as the absence of any movement except for those necessary for respiration, were scored offline using automated analysis with VideoTrack software (ViewPoint, Civrieux, France), as previously described [59,65]. The number of rearings and the duration of grooming were manually scored from the video recordings by an observer blinded to the group allocation. To prevent potential bias from daytime and other factors, animals from all groups were tested in an interchanging order, and four boxes were used in one run.

#### 2.4.2. Conditioned Taste Aversion Test

First, the basal level of preference for a 1% sucrose solution over tap water was determined using a 2-bottle paradigm to exclude a priori group differences in this measure. Animals were given a free choice for 8 h of testing, during the active period of their daily cycle, starting at 9 a.m. We used specialized drinking bottles that ensured minimal spillage of liquid that did not exceed 0.1 mL per 24 h, which is less than 5% of the average liquid intake in C57BL6 mice [31,66,67]. The bottles were weighed at the start and end of the experiment using a balance with a resolution of 0.1 g to calculate consumption, as described elsewhere [31,66,67]. During training, the mice were deprived of water for 21 h and then allowed to drink a freshly prepared 2.5% sucrose solution for 30 min using a one-bottle paradigm. An i.p. injection of lithium chloride (0.24 M) solution prepared ex temporo (Sigma Aldrich, Darmstadt, Germany) at a dose of 2% of body weight and an i.p. injection of 0.9%sodium chloride (NaCl) vehicle was administered thereafter. After the injection, the animals were allowed to drink a 2.5% sucrose solution for 1.5 h.

The following day, the mice were given a choice between tap water and a 1% sucrose solution in a two-bottle paradigm for eight hours, starting at the onset of the active cycle period at 9 am. Bottles were prepared 12 h in advance and kept upside down in the experimental room to ensure temperature balance between solutions and room air and to provide a proper filling of all parts of the bottles. The bottles positions were swapped halfway through the duration of the test to prevent the effects of random side preference in the drinking behavior of mice. Liquid consumption was measured by weighing the bottles before and after each drinking session. Preference for sucrose solution, calculated as the percentage of consumed sucrose versus the total amount of consumed liquid (sucrose preference = [amount of sucrose consumed/amount of sucrose and water consumed] × 100%), was calculated as previously described [31,33]. The absence of a statistical difference from the chance level of 50% in the choice between regular water on the day of recall and sweetened water was considered an indicator of associative memory.

#### 2.4.3. Pellets Displacement Test

The ability to displace small objects, such as small stones or food pellets, from a tube inside the cage is species-specific in mice and depends on an intact hippocampal formation [68]. In this test, 20 standard food pellets were placed into a cardboard tube (diameter = 4 cm, length = 10 cm) that was introduced into an observation transparent cage made of plexiglass (15 × 20 × 30 cm) covered with a small amount of bedding. A new disposable tube was used for each animal to avoid transferring olfactory cues from one animal to another. Animals were not food-deprived at any point before or during the test to preclude a confound of the readouts by feeding motivation. A pellet was scored as displaced when it was located entirely outside the tube. The latency to displace the first pellet and the number of pellets displaced in 15 min intervals of a 90 min test were scored as described elsewhere [22,32,33]. To prevent potential bias from daytime and other factors, animals from all groups were tested in an interchanging order and allowed to acclimatize to a laboratory room for at least 1 h.

#### 2.4.4. Dark Light Box

The dark–light box apparatus (Technosmart, Rome, Italy) consisted of two plexiglass compartments (both 20 × 20 × 25 cm): dark and illuminated (5 lx), connected by a tunnel (5 × 5 × 5 cm). A shadow-free illumination system was used, and sound conditions avoiding noise were considered. The apparatus was surrounded by black curtains in order to reduce reflection and maintain control over lighting conditions during testing. At the beginning of the test, the mice were placed in the dark compartment, from where they could visit the lit box. The 5 min latency to exit the dark compartment, number of exits from the dark compartment, and time spent in the light compartment were scored as described elsewhere [22,31,66]. To prevent potential bias from daytime and other factors, animals from all groups were tested in an interchanging order and allowed to acclimatize to a laboratory room for at least 1 h. The behavior was recorded and scored offline using manual scoring. Experimenter was blind to animal IDs. The apparatus was carefully cleaned between trials.

#### 2.4.5. Elevated O-Maze

The apparatus (Technosmart, Rome, Italy) consisted of a circular path (runway width 5.5 cm, diameter 46 cm), was placed 50 cm above the floor. Two opposing arms were protected by walls (height 10 cm), and the illumination strength was 5 lx. The apparatus was placed on a dark surface and surrounded by black curtains in order to reduce reflection and maintain control over lighting conditions during testing. At the start of the test, mice were placed in one of the closed arm compartments of the apparatus. Latency to enter the open arm, number of entries in the open arm, and time spent in the open arm as indicators of anxiety-like behavior were scored as described elsewhere using previously validated parameters during a 5 min observation period as described elsewhere [22,32]. The apparatus was carefully cleaned between trials. To prevent potential bias from daytime and other factors, animals from all groups were tested in an interchanging order and allowed to acclimatize to a laboratory room for at least 1 h. The behavior was recorded and scored offline using manual scoring. Experimenter was blind to animal IDs.

#### 2.4.6. Step-Down Avoidance

The step-down test is a passive avoidance learning task used to evaluate hippocampus-dependent memory. In this paradigm, animals are trained not to step down from a platform onto a metal grid floor to avoid electric footshocks. The step-down apparatus consisted of a transparent plastic cubicle (25 × 25 × 50 cm) with a stainless-steel grid floor (33 rods, 2 mm in diameter) (Technosmart, Rome, Italy) on which a square wooden platform (7 × 7 × 1.5 cm) was placed on. The animals were allowed to acclimatize to the test room for at least 1 h before the start of the test. The mice were placed on a platform in an opaque cylinder. The illumination strength was 25 lx. In this paradigm, animals are trained not to step down from the platform onto a grid floor to avoid electric shock. During the training session on day 1, the mice were placed on the platform inside a transparent cylinder for 30 s to prevent them from stepping down immediately. After removal of the cylinder, the time until the animal left the platform with all four paws was measured as the baseline latency of the step-down. Immediately after the step-down, the mice received a single alternating electric current (AC, 50 Hz, 0.8 mA, 1 s) and were immediately returned to their cages.

On day 2, during the recall trial session, the mice were returned to the apparatus and placed on a platform inside a transparent cylinder. No foot shocks were administered. After removing the cylinder following a 30 s adaptation period, the latency of step-down with all four paws was measured until 180 s elapsed. This latency was scored as a measure of contextual memory, as previously described [32,69,70]. The test apparatus was illuminated with white light (25 lx) and cleaned thoroughly with 70% ethanol for 10 min before introducing each animal.

#### 2.4.7. Y-Maze

The ability of animals to use spatial cues to find a bottle with drinking water was investigated in the spatial version of the Y-maze model, a paradigm of hippocampus-dependent memory, as described previously [71,72]. The Y-maze was constructed using black Plexiglass and consisted of three arms (40 × 6 × 10 cm) with an angle of 120° between each symmetrical arm (Technosmart, Rome, Italy). Figures of various shapes and colors (approx. size 20 × 40 and 20 × 30 cm) were placed on the walls of the room to allow for spatial orientation. These visual cues cut from paper were placed around the maze approximately 1.5 m. The illumination strength was 5 lx. Validation studies have shown that the 180° rotation of the cues around the Y-maze apparatus disrupts the performance of previously trained mic [71]. At the ends of the arms, two identical small bottles, one filled with water and the other empty, were placed in a position that was adjusted to allow the mice to drink water. Before the first session, the mice were deprived of water for 18 h. The mice were trained in two consequent 10 min trials spaced one hour apart for five days. The latencies for reaching the bottle of water, percentage of correct choices, and drinking duration were recorded.

The experimental groups were randomized to receive water rewards from either the left- or right-hand bottle. Each mouse was allowed to drink for up to 20 min during the training session (and was removed to the home cage when the time elapsed). When no water intake occurred by the end of the training day, free access to water in the home cages was allowed for 20 min, with a delay of at least one-hour post-training. Two 15 min trials per day spaced 3–4 h apart, were carried out for five consecutive days. The intermittent order of testing animals was ensured to minimize the potential effects of daytime and other biases on group comparison. The animals’ body weight was monitored throughout the testing period; previous studies have shown this Y-maze protocol to be optimal for training and demonstrated a lack of negative effects of the drinking schedule on body weight [71,72]. The latency to reach the filled bottle and the percentage of correct choices for the arm containing this bottle were used as indicators of learning the task. The animals were acclimatized to the experimental room for at least 1 h prior to the assay, and the apparatus was carefully cleaned between sessions. Newly cleaned bottles were used in each session.

#### 2.4.8. Pole Test

Motor coordination was assessed using the pole test. Mice were placed individually on the top of a vertically oriented wooden bar (diameter, 1.1 cm; height, 60 cm) and allowed to descend the bar toward a horizontal surface. The latency to descend the pole and reach the horizontal surface was recorded. In addition to descent latency, the animals’ performance was visually monitored for abnormal descent behavior. A markedly reduced latency associated with sliding down the pole, rather than controlled stepping, was considered indicative of impaired motor coordination and was scored as a motor deficit. Before testing, mice were transferred to the laboratory room at least 1 h in advance to allow acclimatization to the experimental environment. Testing was conducted during the dark phase of the animals’ light/dark cycle. To minimize potential bias associated with testing order, animals from different experimental groups were tested in an interleaved sequence. All tests were performed by the same experimenter, who was blinded to the animals’ genotype and treatment as described elsewhere [53,54].

#### 2.4.9. Wire Test

The mice were placed on a horizontal wire (diameter, 0.3 cm; height above the surface, 60 cm) and allowed to grip the wire for up to 180 s. The latency to fall was recorded as a measure of motor strength, coordination, and grip performance. Before testing, mice were transferred to the laboratory room at least 1 h in advance to allow acclimatization to the experimental environment. Testing was conducted during the dark phase of the animals’ light/dark cycle. To minimize potential bias associated with testing order, animals from different experimental groups were tested in an interleaved sequence. All tests were performed by the same experimenter, who was blinded to the animals’ genotype and treatment as described elsewhere [54].

#### 2.4.10. Rotarod Test

Motor coordination and endurance were assessed using a rotarod apparatus (Columbus Instruments, Columbus, OH, USA). Mice were placed individually on the rotating rod, maintained at a constant speed of 15 rpm, and tested for a maximum duration of 600 s. The latency to fall from the rod was recorded for each animal and used as a measure of motor performance, with longer latencies indicating better motor coordination and endurance as described elsewhere [53,54]. Before testing, animals were transferred to the experimental room at least 1 h in advance to allow acclimatization to the laboratory environment. To familiarize the animals with the apparatus, mice were placed on the rotating rod for 10 s on three consecutive occasions. Testing was conducted during the dark phase of the animals’ light/dark cycle. To minimize potential bias associated with testing order, animals from different experimental groups were tested in an interleaved sequence. All tests were performed by the same experimenter, who was blinded to the animals’ genotype and treatment.

### 2.5. Culling of Mice and Tissue Collection

Mice were euthanized under terminal anesthesia induced by isoflurane (Sigma-Aldrich, Saint Louis, MO, USA) in an anesthesia machine (RWD Life Science Co., Guangdong, China) according to a previously established protocol [73]. After perfusion with 20 mL of PBS, one hemisphere was removed, and perfusion with 20 mL of 4% paraformaldehyde solution (pH 7.4) (Sigma-Aldrich, Saint Louis, MO, USA) was continued [73]. In FUS[1-359]-Tg mice, the lumbar spinal cord was collected after perfusion, as described elsewhere [54]. Tissues were post-fixed in 4% paraformaldehyde solution at +4 °C overnight, as described elsewhere [73], and stored until further use in immunofluorescence staining.

### 2.6. Histological Analysis and Sectioning of Brains

After fixation, the tissues were washed 3 times with PBS for one hour and then dehydrated using graded ethanol solutions (75% for 1 h, 95% (I) for 5 min, 95% (II) for 5 min, 95% (III) for 5 min, 100% (I) for 5 min, and 100% (II) for 10 min). Each sample was incubated consecutively with 100% ethanol–chloroform (1:1) for 30 min, chloroform (I) for one h, and chloroform (II) overnight, and embedded in Surgipath Tissue Infiltration Medium (Leica Biosystems Inc., Nissloch, Germany) (three times, for one hour each) at 60 °C, using a Leica EG1160 tissue embedding station (Leica Biosystems, Nissloch, Germany). For the analysis, a 400-µm-wide zone of the hemisphere was examined, with the starting point defined at 1100 µm lateral to the midline. Every 10th section was mounted on polylysine-coated slides (Thermo Fisher Scientific Inc., Kalamazoo, MI, USA), 8-µm-thick sections were analyzed, with 80 µm between consecutive analyzed sections [30,32,33,74].

### 2.7. Histological Analysis and Sectioning of Spinal Cords

Spinal cord histological sections were prepared and mounted as described previously [35,54]. Lumbar regions of spinal cords were washed from fixative in PBS, 3 washes of 10 min and then dehydrated using graded ethanol solutions (75% for 30 min, 95% (I) for 5 min, 95% (II) for 5 min, 95% (III) for 5 min, 100% (I) for 5 min, and 100% (II) for 10 min). Each sample was incubated consecutively with 100% ethanol–chloroform (1:1) for 30 min, chloroform (I) for 30 min, and chloroform (II) overnight, and embedded in Surgipath Tissue Infiltration Medium (Leica Biosystems Inc., Nissloch, Germany) (three times, for one hour each) at 60 °C, using a Leica EG1160 tissue embedding station (Leica Biosystems, Nissloch, Germany). Spinal cords were then sectioned into 8-μm-thick sections and every 10th section was mounted on polylysine-coated slides (Thermo Fisher Scientific Inc., Kalamazoo, MI, USA). Cohorts of mice containing five animals in the control WT group and seven animals in groups of mutants (each group was composed of seven mice) were randomized according to body weight changes and the manifestation of paralysis to balance the parameters with values of all mice used in the study (Supplementary Materials, Figure S2). The remaining mice (seven in each of the three groups) were used in additional biochemical assays that are not presented in this study.

### 2.8. Congo Red Staining and Imaging of Amyloid Plaques

For amyloid plaque staining, brain sections were deparaffinized in xylene for 20 min, rehydrated using graded ethanol solutions (100% for 20 min, 95 stained % for 5 min, and 50% for 5 min), washed three times with deionized water for 5 min, with 0.5% Congo red (Sigma-Aldrich, Saint Louis, MO, USA) solution in 50% ethanol for 5 min, and differentiated using 0.2% KOH in 80% ethanol for 3 min. The slices were then embedded in the ImmuMount water-based mounting medium (Thermo Fisher Scientific Inc., Kalamazoo, MI, USA). Ten slices per animal were analyzed using confocal laser scanning microscopy (LSM880; Carl Zeiss, Oberkochen, Germany) in the tile scan mode.

**Figure 2:**
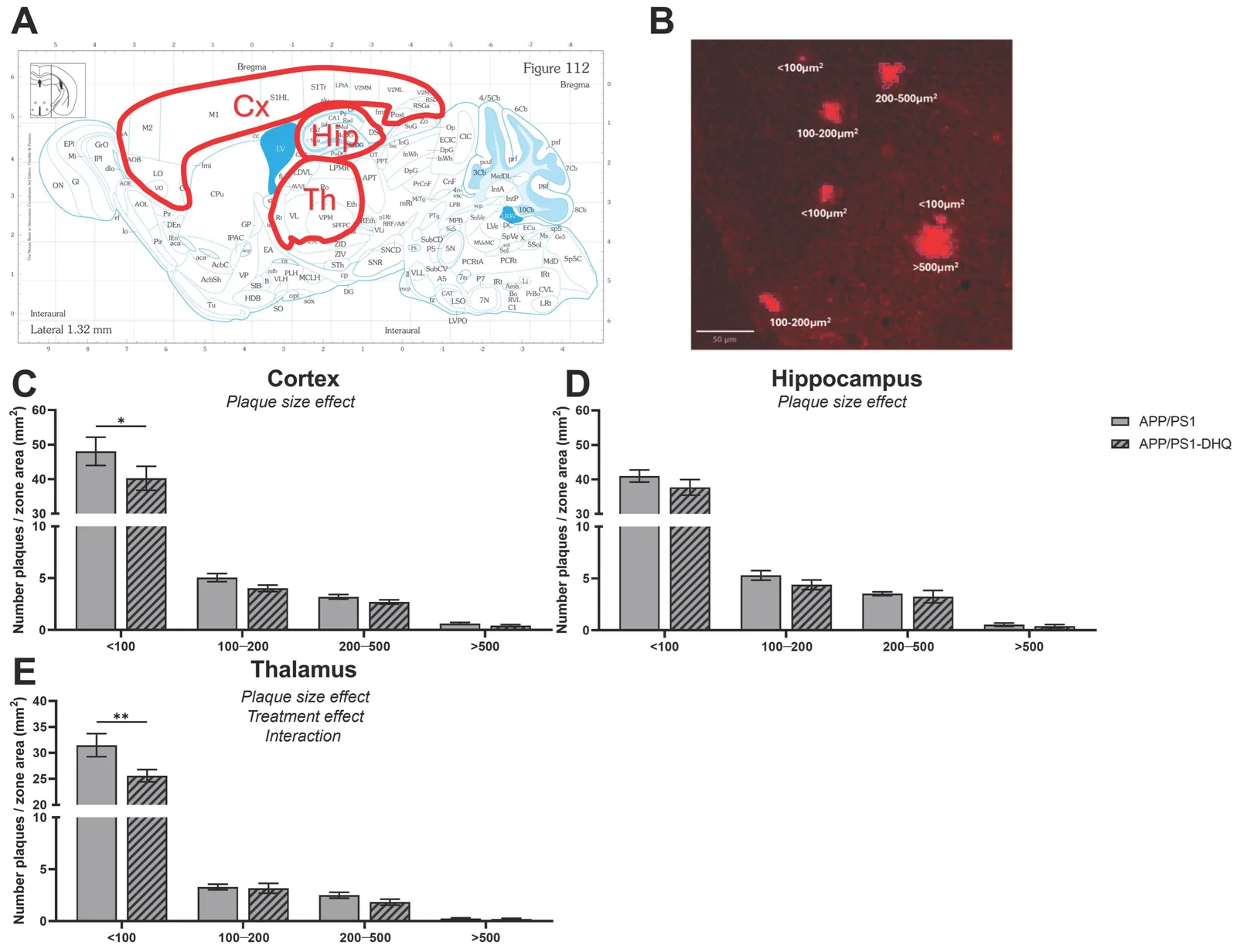
Effect of DHQ administration on amyloid plaque density in the brains of APP/PS1 mice. (**A**) Schematic representation of the analyzed regions: cortex (Cx), hippocampus (Hip), and thalamus (Th). (**B**) Amyloid plaques were visualized using Congo red staining. Representative image illustrating plaques of different sizes in the cortex of APP/PS1 mice. Plaques were categorized according to size (<100, 100–200, 200–500, and >500 µm^2^). Scale bar is 50 μm. (**C**) Amyloid plaque density in the cortex. (**D**) Amyloid plaque counts in the hippocampus. (**E**) Amyloid plaque counts in the thalamus. * *p* < 0.05, ** *p* < 0.01; two-way ANOVA and Sidak’s multiple comparisons test; data are presented as mean ± SEM. DHQ—dihydroquercetin. Investigated brain structures are outlined in red.

Three regions of interest (ROIs)–the cortex, hippocampus, and thalamus–were outlined in accordance with the mouse brain atlas (Figure 2A) [75]. For the visualization of brain morphology, Congo red staining was paired with transmitted-light photomultiplier (T-PMT) imaging. Amyloid plaques were detected in each ROI in the Congo red channel by intensity thresholding using the QuPath software, version 0.4.3 (Belfast, Northern Ireland, UK) [31,32,33]. Because slight variations in staining intensity can produce small differences in the background signal, a single fixed threshold could not be applied to the entire dataset. Instead, the threshold was manually set for each image in the range of 60–80 (on an intensity scale of 0–255), chosen as a value that separated the plaque-related signal from the local background tissue signal in that section. The area of each retained plaque was measured, and plaques were assigned to four size classes: <100, 100–200, 200–500 and >500 µm^2^. For each class, plaque counts were normalized to the annotated ROI area of the same section and averaged across ten sections to obtain a single density value (plaques/mm^2^) per region per animal, as described elsewhere [76]. Image files were coded with random numeric identifiers by an investigator not involved in the quantification, and groups were revealed only after all measurements were completed.

### 2.9. Immunofluorescent Staining of Activated Astrocytes

Brain sections were incubated with primary anti-GFAP antibody (1:1000, ab7260, Abcam, Waltham, MA, USA) at +4 °C overnight, followed by incubation with secondary goat anti-rabbit IgG (H + L) antibodies (1:1000, A11011, Alexa Fluor 568, Thermo Fisher Scientific, Waltham, MA, USA). The nuclei were counterstained with DAPI (1:1000, 62248; Thermo Fisher Scientific Inc., Kalamazoo, MI, USA) and embedded in an ImmuMount water-based mounting medium (Thermo Fisher Scientific Inc., Kalamazoo, MI, USA). The area of GFAP-positive astrocytes was measured using QuPath software, version 0.4.3 (Belfast, Northern Ireland, UK) [33,76,77]. The same image analysis parameters were applied to all images in QuPath, including Gaussian smoothing with a smoothing sigma of 0.5. The thresholds ranged from 140 to 150. Anatomical regions of interest were manually delineated in QuPath based on DAPI staining of cell nuclei and the anatomical boundaries defined by the Mouse Brain Atlas (Figure 2A) [75]. Four slices per animal were analyzed, and the cell density was calculated separately for each section and then averaged across sections to obtain a single value for each animal.

### 2.10. Nissl Staining of Motor Neurons

Nissl staining of transverse sections of the lumbar region (L3–L5) of the spinal cord was performed as previously described [35,54]. Motor neurons were counted based on the presence of large cell bodies containing Nissl granules, a clear nuclear envelope, and an intensely stained nucleolus in six sections separated by 80 µm intervals. The average number from six sections per spinal cord region per animal was used for statistical analysis.

### 2.11. Malondialdehyde Assay

MDA content was measured in the prefrontal cortex, which was dissected on dry ice and stored at −80 °C until analysis. First, the tissue was weighed and homogenized in 1 mL of ice-cold 0.01 M PBS (pH 7.2) using a handheld rotor-stator homogenizer TissueRuptor II (QIAGEN Sciences Inc., Germantown, MD, USA). Homogenates were centrifuged at 1000× *g* for 20 min at 4 °C, and the supernatants were collected. MDA levels were quantified using a competitive inhibition ELISA kit (CEA597Ge, Cloud-Clone Corp., Katy, TX, USA) according to the manufacturer’s instructions. Standards and samples were assayed in duplicate on the same plate, absorbance was read at 450 nm, and concentrations were interpolated from a five-point standard curve. MDA values were normalized to the wet weight of the corresponding tissue sample and are expressed as ng per mg of tissue.

### 2.12. Statistical Analysis

Statistical analyses were performed using GraphPad Prism software (version 8.0; GraphPad Software, San Diego, CA, USA). No criteria were set for the inclusion or exclusion of the animals. The normality of the distribution was assessed using the Shapiro–Wilk test. For data exhibiting a normal distribution, comparisons between the two groups were conducted using the *t*-test, and the Mann–Whitney U-test was used for non-normally distributed data. Comparisons between more than two normally distributed groups were examined using one-way, two-way, or three-way analysis of variance (ANOVA), followed by Tukey’s test. A sensitivity power analysis was performed using G*Power 3.1.9.7 to determine the minimum effect size detectable with the achieved group sizes. At α = 0.05 and 80% power, the APP/PS1 comparison (*n* = 5–6 per group) was able to detect effects of Cohen’s d ≥ 1.9, the WT comparison (*n* = 9 per group) effects of d ≥ 1.4, and the FUS–vehicle vs. FUS–DHQ comparison (*n* = 14 per group) effects of d ≥ 1.3 after adjustment for multiple comparisons. These thresholds are appropriate for detecting the large-magnitude effects expected in these models.

For comparisons among several groups, analysis of variance (ANOVA) was performed, followed by Tukey’s honestly significant difference (HSD) post hoc test, which is specifically designed to address multiple pairwise comparisons by controlling the family wise error rate (FWER). This test enables the adjustment of necessary corrections for multiple comparisons to avoid false-positive results and the probability of making one or more Type I errors across the entire set of comparisons within each ANOVA model. Repeated measurements (RM) in the conditioned taste aversion, Y-maze, step-down test results were analyzed using three-way repeated-measures ANOVA, and pellet displacement results were analyzed using two-way repeated-measures ANOVA. For Y-maze and pellet displacement results Greenhouse–Geisser correction was applied to account for the violation of the sphericity assumption. In the conditioned taste aversion and step-down avoidance tests each animal was measured only twice, so the correction has no effect and was not applied. For data with more than two groups that were not normally distributed, the Kruskal–Wallis test with post hoc Dunn test was used. The percentage of mice exhibiting ALS symptoms and the percentage of correct trials in the Y-maze test were compared using Fisher’s exact test. Differences in sucrose preference from chance levels in the conditioned taste aversion test were analyzed using a one-sample *t*-test. No data points were excluded from the analysis. The level of significance was set at *p* < 0.05. The results are presented as mean ± standard error of the mean (SEM).

## 3. Results

### 3.1. DHQ Administration Decreased Number of Small Amyloid Plaques in the Cortex and Thalamus of APP/PS1 Mice

Amyloid plaques were analyzed in three brain regions: the cortex, hippocampus, and thalamus (Figure 2A). Plaques stained with Congo red were categorized according to their size (<100 µm^2^, 100–200 µm^2^, 200–500 µm^2^, and >500 µm^2^). In the cortex, a significant main effect of plaque size was observed (F = 195.3, *p* < 0.0001, two-way ANOVA), while neither treatment (F = 2.566, *p* = 0.1163, two-way ANOVA) nor the interaction (F = 1.488, *p* = 0.2308, two-way ANOVA) between treatment and plaque size was significant. Post hoc Sidak’s multiple comparisons test revealed a significant reduction in the number of small plaques (<100 µm^2^) in DHQ–treated compared with untreated APP/PS1 mice (*p* = 0.0469), whereas larger plaque categories were not affected by the treatment (Figure 2C).

In the hippocampus, plaque size significantly influenced plaque abundance (F = 599.8, *p* < 0.0001, two-way ANOVA), but neither treatment (F = 2.369, *p* = 0.1310, two-way ANOVA) nor the interaction between factors (F = 0.9434, *p* = 0.4279, two-way ANOVA) reached statistical significance, and no differences were detected between groups across plaque size categories (Figure 2D).

In the thalamus, both plaque size (F = 302.7, *p* < 0.0001, two-way ANOVA) and treatment (F = 4.776, *p* = 0.0342, two-way ANOVA) significantly affected plaque counts, and a significant interaction between the factors was detected (F = 3.350, *p* = 0.0273, two-way ANOVA) (Figure 2E). The number of small amyloid plaques (smaller than 100 µm^2^) was significantly reduced in DHQ-treated APP/PS1 mice compared to that in non-treated APP/PS1 mice (*p* = 0.0016, Sidak‘s test).

### 3.2. Immunohistochemical Analysis of Activated Astrocytes and Evaluation of Malondialdehyde Brain Levels

In the cortex of the investigated groups of mice, only genotype had a significant effect (F = 48.57, *p* = 0.0001, two-way ANOVA) on the area of GFAP-positive cells, with no treatment (F = 0.1723, *p* = 0.6890, two-way ANOVA) or genotype × treatment interaction (F = 0.01688, *p* = 0.8998, two-way ANOVA) reaching significance. The area of GFAP-positive cells was significantly increased in both untreated and DHQ-treated APP/PS1 mice compared to the respective WT groups (*p* = 0.0045 and *p* = 0.0057, respectively, Sidak’s test).

In the hippocampus, a significant difference in the area of GFAP-positive cells was determined by the genotype effect (F = 32.65, *p* = 0.0004, two-way ANOVA), with no treatment (F = 1.106, *p* = 0.3237, two-way ANOVA) or genotype × treatment interaction (F = 1.001, *p* = 0.3463, two-way ANOVA) reaching significance. The area of GFAP-positive cells was significantly higher in APP/PS1 mice than in both untreated (*p* = 0.0416, Sidak’s test) and DHQ-treated WT mice (*p* = 0.0063, Sidak’s test).

**Figure 3:**
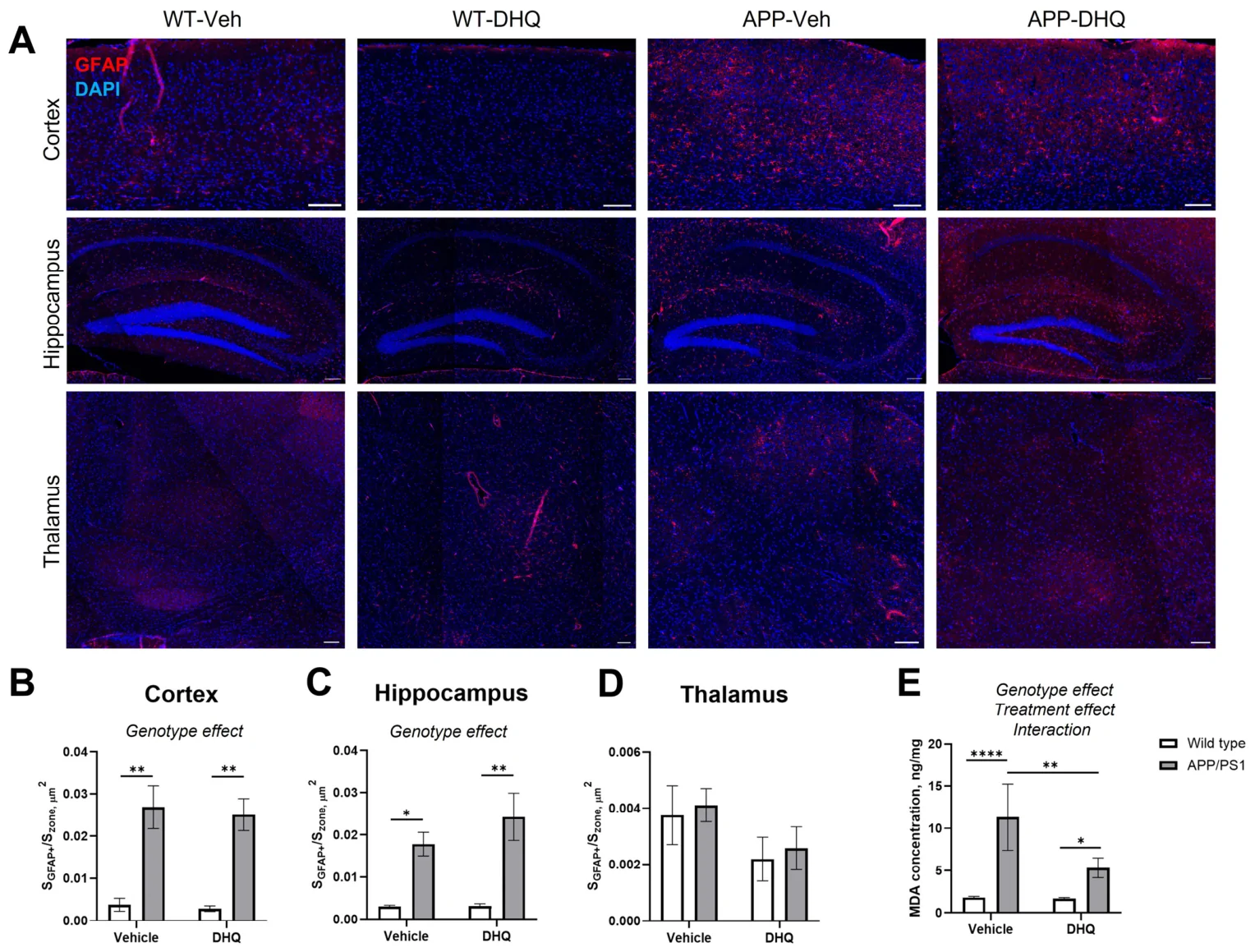
Immunohistochemical analysis of activated astrocytes and changes in cortical malondialdehyde levels. (**A**) Immunofluorescent images of stained brains of WT and APP/PS1 mice treated with vehicle or DHQ with anti-GFAP (red signal) antibodies, labeling astrocytes, and DAPI staining (blue signal), labeling nuclei. (**B**) Area of GFAP-positive cells in the cortical region. (**C**) Area of GFAP-positive cells in the hippocampus. (**D**) Area of GFAP-positive cells in the thalamus. (**E**) Malondialdehyde concentration in the cortex, measured using competitive ELISA. * *p* < 0.05, ** *p* < 0.01, **** *p* < 0.0001, two-way ANOVA and post hoc Tukey’s test, *n* = 5–9. Data are presented as mean ± SEM. DHQ—dihydroquercetin, WT—wild type.

Two-way ANOVA revealed significant genotype (F = 25.14, *p* < 0.001), treatment (F = 5.427, *p* = 0.0294), and genotype × treatment interaction (F = 4.982, *p* = 0.0361) effects on brain MDA concentration. Post hoc test indicated that significantly higher MDA levels were observed in the untreated APP/PS1 group compared to those in the untreated WT group (*p* = 0.0004, Tukey’s test). The DHQ-treated APP/PS1 group had significantly lower MDA levels than the untreated APP/PS1 group (*p* = 0.0183, Tukey’s test). At the same time, DHQ-treated APP/PS1 mice showed higher MDA levels than those in DHQ-treated WT mice (*p* = 0.0398, Tukey’s test; Figure 3E).

### 3.3. Effects of DHQ on Emotionality Scores of the APP/PS1 Mice

**Figure 4:**
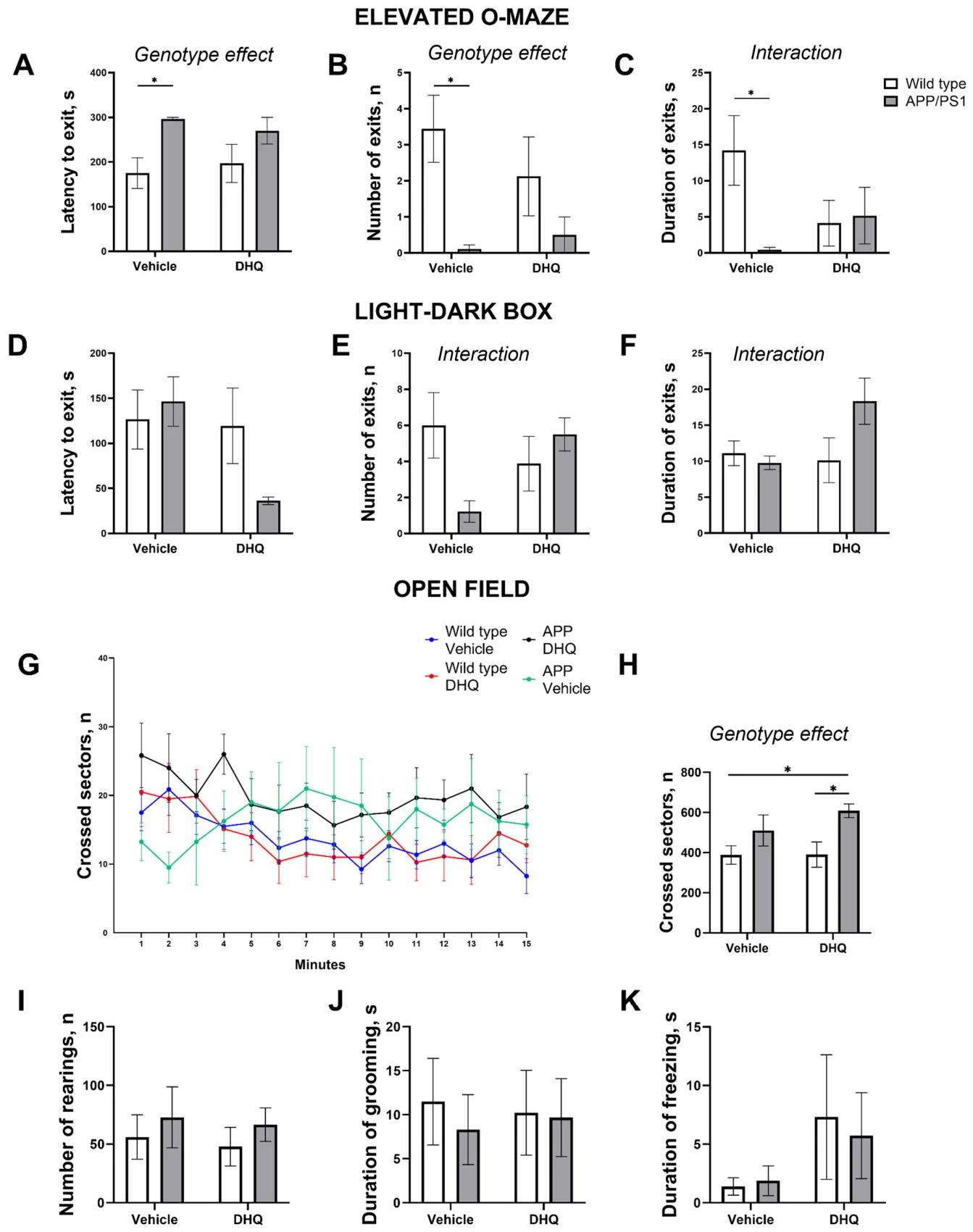
Emotionality scores of APP/PS1 mice and WT mice following DHQ administration. (**A**) Latency to exit the closed compartment of the O-maze. (**B**) Number of exits in the open compartment of the O-maze. (**C**) Time spent in the O-maze open compartment. (**D**) Latency to exit the dark compartment of the LDB. (**E**) Number of exits into the LDB light compartment. (**F**) Time spent in the light compartment of the LDB. (**G**) Number of Open Field sector crosses during 15 min of open field test. (**H**) Total number of crossed sectors. (**I**) Number of rearing. (**J**) Time spent grooming the animal. (**K**) Time spent freezing. * *p* < 0.05, two-way ANOVA and post hoc Tukey’s test, *n* = 5–9. Data are presented as mean ± SEM. DHQ—dihydroquercetin, WT—wild type.

In the elevated O-maze test, two-way ANOVA revealed a significant genotype effect (F = 9.554, *p* = 0.0045) without a significant treatment effect (F = 0.005807, *p* = 0.9398) or genotype × treatment interaction (F = 0.5851, *p* = 0.4507) for the latency to exit to the open arm. This measure was significantly higher in untreated APP/PS1 mice than in untreated WT mice (*p* = 0.0318, Tukey’s test; Figure 4A). A significant genotype effect (F = 9.743, *p* = 0.0042, two-way ANOVA) was observed, but no significant treatment effect (F = 0.343, *p* = 0.562, two-way ANOVA) or genotype × treatment interaction (F = 1.15, *p* = 0.291, two-way ANOVA) was found for the total number of exits. This parameter was significantly lower in untreated APP/PS1 mice than in untreated WT mice (*p* = 0.0173, Tukey’s test; Figure 4B). A significant genotype × treatment interaction (F = 4.403, *p* = 0.0450, two-way ANOVA) was demonstrated for the total duration of exits, while the main effects of genotype (F = 3.252, *p* = 0.0821, two-way ANOVA) and treatment (F = 0.5793, *p* = 0.4530, two-way ANOVA) were not significant. This indicator was significantly reduced in untreated APP/PS1 mice compared to that in untreated WT mice (*p* = 0.0293, Tukey’s test; Figure 4C).

In the dark light box, the treatment effect demonstrated a strong trend for latency to exit (F = 3.256, *p* = 0.0819, two-way ANOVA; Figure 4D) without a significant genotype effect (F = 0.9497, *p* = 0.3381) or genotype × treatment interaction (F = 2.518, *p* = 0.1238, two-way ANOVA). Two-way ANOVA revealed a significant genotype × treatment interaction (F = 4.395, *p* = 0.0452) for the time spent in the light zone, whereas effects of genotype (F = 2.282, *p* = 0.1421) and treatment (F = 2.766, *p* = 0.1074) were not significant. A strong trend for an increase in this parameter was observed in the DHQ-treated APP/PS1 group compared to the DHQ-treated WT group (*p* = 0.0726, Tukey’s test; Figure 4F). A significant genotype × treatment interaction was detected for the total number of exits (F = 5.345, *p* = 0.0284, two-way ANOVA), with no significant genotype (F = 1.296, *p* = 0.2646, two-way ANOVA) or treatment effects (F = 0.6042, *p* = 0.4435, two-way ANOVA). A strong trend for a decrease in this parameter was shown in the untreated APP/PS1 group compared to the untreated WT group (*p* = 0.0631, Tukey’s test; Figure 4E).

In the open field test, the genotype effect was significant (F = 9.995, *p* < 0.0001, two-way ANOVA, Figure 4G). For the number of crossed sectors scored per minute, a significant genotype effect was revealed (F = 8.591, *p* = 0.0077, two-way ANOVA) without a significant treatment effect or genotype × treatment interaction (F = 0.738, *p* = 0.399 and F = 0.684, *p* = 0.417, respectively, two-way ANOVA). The total number of crossed sectors was significantly higher in DHQ-treated APP/PS1 mice than in DHQ-treated WT mice (*p* = 0.0437, Tukey’s test; Figure 4H). No significant genotype effect, treatment effect or genotype × treatment interaction was found for total number of rearings (F = 0.842, *p* = 0.368; F = 0.14, *p* = 0.711 and F = 0.0026, *p* = 0.959, respectively; Figure 4I), total duration of grooming events (F = 0.134, *p* = 0.717; F = 0.0001, *p* = 0.991 and F = 0.0066, *p* = 0.799, respectively; Figure 4J), and total duration of freezing events (F = 0.0198, *p* = 0.889; F = 1.543, *p* = 0.227 and F = 0.007, *p* = 0.793, respectively; Figure 4K).

### 3.4. Effects of DHQ on Learning of APP/PS1 and WT Mice

**Figure 5:**
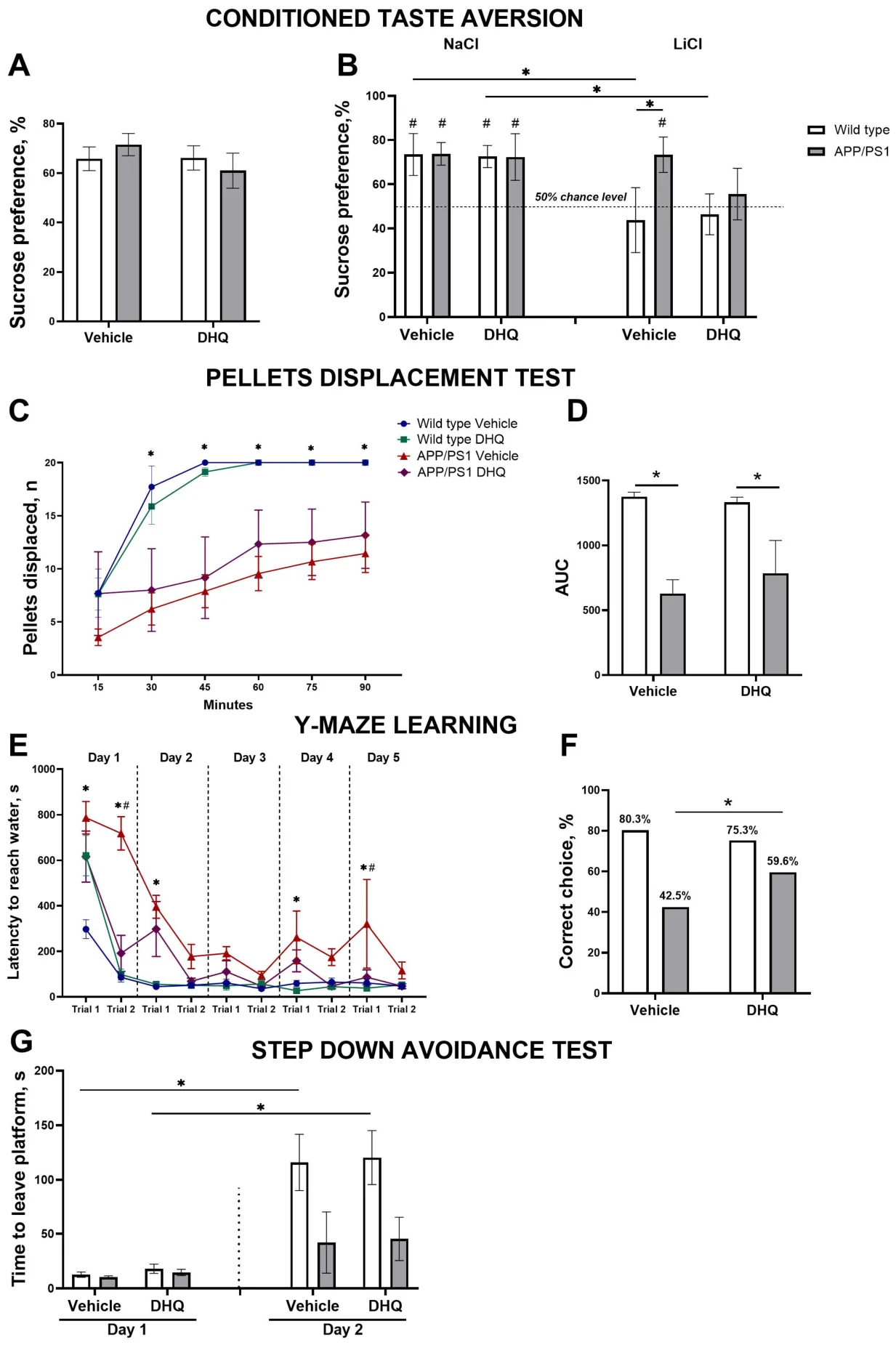
Effects of DHQ on learning in APP/PS1 and WT mice (**A**) Comparison of sucrose and water consumption in the sucrose preference test. (**B**) Sucrose preference in the conditioned taste aversion test. (**C**) Number of displaced pellets in the pellet displacement test, plotted in 15 min intervals. (**D**) AUC for the cumulative time-course plot of displaced pellets. (**E**) Latency to reach the water reward in the Y-maze by day. (**F**) Average percentage of correct choices in the Y-maze. (**G**) Latency to step down in the step-down avoidance test. * *p* < 0.05, two-way ANOVA, two-way or three-way repeated measures ANOVA and post hoc Tukey’s test, Fisher’s exact test, # *p* < 0.05 vs. chance level, one-sample *t*-test, *n* = 5–9. Data are presented as mean ± SEM. DHQ—dihydroquercetin, AUC—area under curve. The vertical dotted line indicates a break separating measurements taken on Day 1 and Day 2.

No significant genotype effect (F = 0.002714, *p* = 0.9589, two-way ANOVA), treatment effect (F = 0.8312, *p* = 0.3706, two-way ANOVA), or genotype × treatment interaction (F = 0.9354, *p* = 0.3427, two-way ANOVA; Figure 5A) was observed for baseline sucrose preference (Figure 5A). A significant day effect (F = 35.24, *p* < 0.0001, three-way RM ANOVA) and genotype effect (F = 9.947, *p* = 0.0002, three-way RM ANOVA), as well as their interaction (F = 9.897, *p* = 0.0151, three-way RM ANOVA) and genotype × treatment interaction (F = 2.896, *p* = 0.0236, three-way RM ANOVA) were observed for sucrose preference in the recall session of the conditioned taste avoidance test. A strong trend was observed for the treatment effect (F = 2.024, *p* = 0.0534, three-way RM ANOVA) without significant treatment × day or treatment × genotype × day interactions (F = 1.093, *p* = 0.3836 and F = 2.615, *p* = 0.1814, respectively, three-way RM ANOVA). This parameter was significantly lower in both untreated and DHQ-treated WT animals following LiCl injection (*p* = 0.0049 and *p* = 0.0152, respectively; Tukey’s test). At the recall session, sucrose preference was significantly lower in the untreated WT group than in the untreated APP/PS1 group (*p* = 0.0002, Tukey’s test; Figure 5B). Following LiCl challenge, only the untreated APP/PS1 group had a significantly higher sucrose preference than the chance level, but not the DHQ-treated mice (*p* = 0.0028 and *p* = 0.347, one-sample *t*-test; Figure 5B). The number of animals displaying > 50% sucrose preference was significantly higher in untreated mutants than in the DHQ-treated WT group, indicating a learning deficit in the former group (*p* < 0.0445, Fisher’s test).

In the pellet displacement test, no significant genotype effect (F = 0.006526, *p* = 0.8010, two-way ANOVA), treatment effect (F = 2.233, *p* = 0.1507, two-way ANOVA), or genotype × treatment interaction (F = 0.2381, *p* = 0.6309, two-way ANOVA) was observed for latency to displace the first pellet. Significant genotype effect in the number of displaced pellets, scored in 15 min intervals, was revealed (F = 8.960, *p* = 0.0003, two-way RM ANOVA), with no significant treatment effect (F = 0.2269, *p* = 0.6378, two-way RM ANOVA) and genotype × treatment interaction (F = 0.6569, *p* = 0.4250, two-way RM ANOVA, Figure 5C). This measure was significantly higher in untreated WT mice than in untreated APP/PS1 mice at 30 min (*p* < 0.0001, Tukey’s test), 45 min (*p* < 0.0001, Tukey’s test), 60 min (*p* = 0.0003, Tukey’s test), 75 min (*p* = 0.0017, Tukey’s test), and 90 min (*p* = 0.0050, Tukey’s test). Comparison of DHQ-treated and vehicle-treated APP/PS1 mice revealed no significant difference in the number of pellets displaced at any time point during the test: at 15 min (*p* = 0.4081, Tukey’s test), at 30 min (*p* = 0.9076, Tukey’s test), at 45 min (*p* = 0.9628, Tukey’s test), at 60 min (*p* = 0.7204, Tukey’s test), at 75 min (*p* = 0.8997, Tukey’s test) and at 90 min (*p* = 0.9151, Tukey’s test; Figure 5C). A significant genotype effect (F = 27.46, *p* < 0.0001, two-way ANOVA) without significant treatment effect or genotype × treatment interaction (F = 0.226, *p* = 0.637 and F = 0.657, *p* = 0.425, respectively) was shown for AUC for the cumulative time-course plot of displaced pellets. This measure was significantly lower in vehicle- and DHQ-treated APP/PS1 mice compared to respective controls (*p* = 0.0001 and *p* = 0.0055, respectively, Tukey’s test; Figure 5D). No significant differences were found between vehicle- and DHQ-treated APP/PS1 mice in this measure (*p* = 0.3755, Tukey’s test).

In the Y-maze, three-way RM ANOVA revealed significant effects of genotype (F = 45.30, *p* < 0.0001), treatment (F = 8.29, *p* = 0.0087) and trial (F = 25.44, *p* < 0.0001), together with significant genotype × treatment interaction (F = 8.61, *p* = 0.0077) and trial × genotype interaction (F = 2.84, *p* = 0.0152) interactions. The trial × treatment and trial × treatment x genotype interactions were not significant (F = 1.76, *p* = 0.119 and F = 0.8, *p* = 0.562, respectively, three-way RM ANOVA). This indicator was significantly higher in DHQ-treated WT mice than in the untreated WT group on day 1 of trial 1 (*p* < 0.0001, Tukey’s test, Figure 5E). It was significantly longer in the untreated APP/PS1 group than in the untreated WT group in trials 1 and 2, day 1 (both *p* < 0.0001, Tukey’s test), trial 1 day 2 (*p* < 0.0001, Tukey’s test), trial 1 day 4 (*p* = 0.02, Tukey’s test), and trial 1 day 5 (*p* = 0.0013, Tukey’s test). The latency to reach the water reward was significantly shorter in DHQ-treated APP/PS1 mice than in the untreated APP/PS1 group on trial 2, day 1 (*p* < 0.0001, Tukey’s test) and trial 1, day 5 (*p* = 0.0081, Tukey’s test; Figure 5E). The percentage of correct choices was significantly higher among DHQ-APP/PS1 mice than among untreated mutants (*p* = 0.0233, Fisher’s test, Figure 5F).

Three-way RM ANOVA revealed a significant day effect (F = 25.14, *p* < 0.0001) and genotype effect (F = 8.375, *p* = 0.0082), as well as their interaction (F = 7.206, *p* = 0.0123) for latency to step down. The effects of treatment, treatment × genotype, treatment × day, and genotype × day × treatment interactions were not significant (F = 0.105, *p* = 0.749; F = 0.001, *p* = 0.972; F = 0.001, *p* = 0.996; and F < 0.001, *p* = 0.993, respectively; three-way RM ANOVA; Figure 5G). This measure was significantly increased on day 2 in both untreated and DHQ-treated WT animals compared to day 1 results (*p* = 0.0017 and *p* = 0.0019, respectively, Tukey’s test), but not in untreated and DHQ-treated APP/PS1 groups, suggesting impaired learning in mutants (*p* = 0.989 and *p* = 0.983, respectively, Tukey’s test; Figure 5G).

### 3.5. DHQ Administration Counteracts Neuronal Loss in the Spinal Cord in FUS[1-359]-Tg Mice

**Figure 6:**
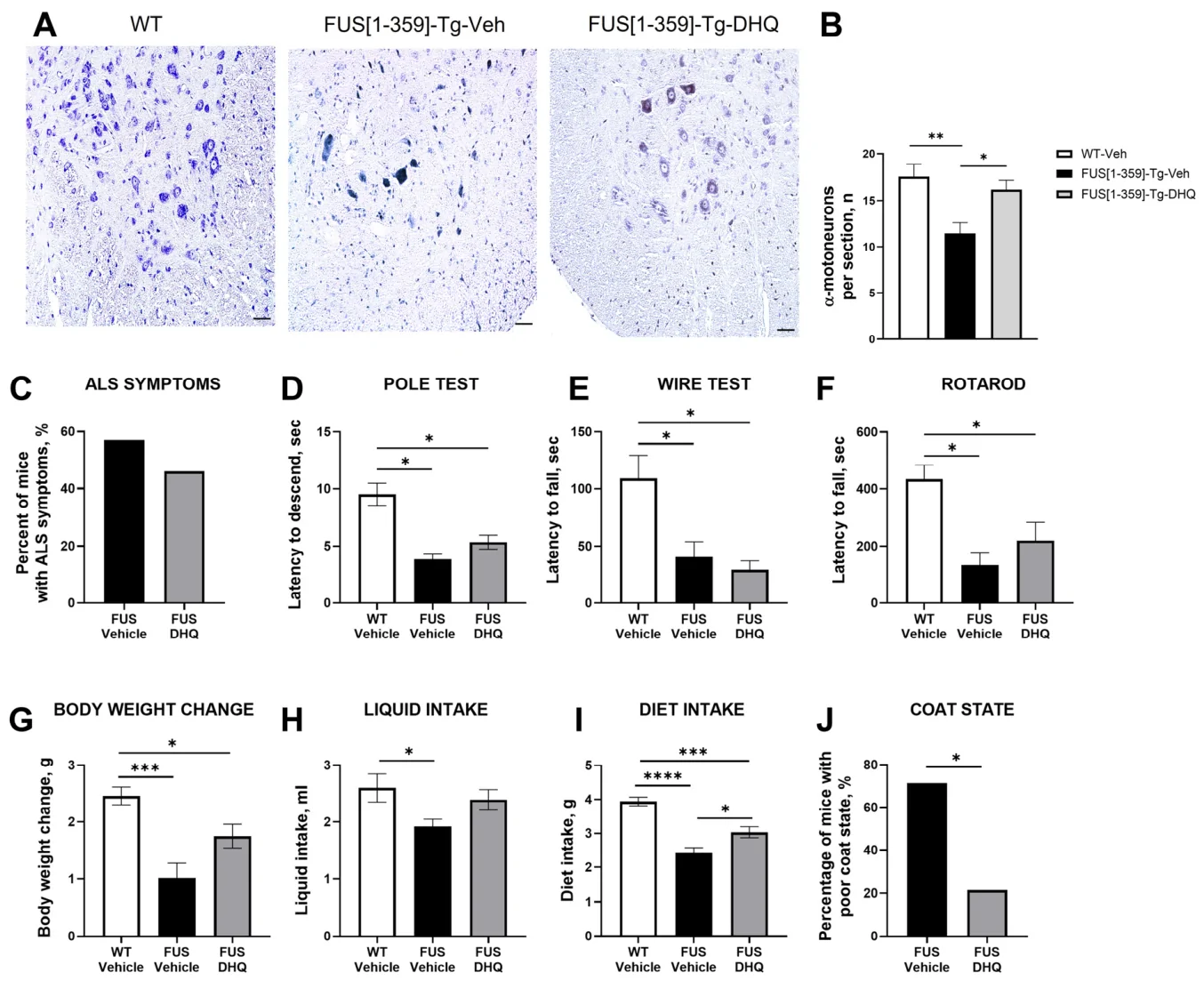
Effect of DHQ administration on the progression of ALS symptoms and spinal cord counts in FUS[1-359]-Tg and WT mice. (**A**) Representative light microscopy images of spinal cord sections of WT (vehicle), FUS[1-359]-Tg (vehicle), FUS[1-359]-Tg (DHQ) mice stained with cresyl violet (Nissl), 20x. Scale bar 50 µm. (**B**) Quantification of lower motor neurons in the ventral horns of each spinal cord section. (**C**) Percentage of mice with ALS symptoms at 105 days of age. Fisher’s exact test. (**D**) Latency to descend in the pole test. (**E**) Latency to fall in the wire test. (F) Latency to fall from the rotarod. (**G**) Body weight changes. (**H**) Average liquid intake of mice. (**I**) Average dietary intake. (**J**) Percentage of mice with poor coat state. (* *p* < 0.05, ** *p* < 0.01, *** *p* < 0.001, **** *p* < 0.0001, one-way ANOVA and post hoc Tukey’s test, Fisher’s exact test, *n* = 12–14 for behavioral analysis, *n* = 5–7 for histological assay. Data are presented as mean ± SEM. DHQ—dihydroquercetin, WT—wild type, FUS—fused in sarcoma.

Nissl staining of spinal cord sections exhibited normal morphology in wild-type mice, with notable morphological alterations in FUS[1-359]-Tg mice, characterized by shrunken cell bodies and less defined nuclei (Figure 6A). Quantitative analysis revealed a significant difference in neuronal density between the experimental groups (F = 6.435, *p* = 0.0096, one-way ANOVA). Statistically significant loss of cells was demonstrated in untreated FUS[1-359]-Tg mice in comparison to untreated WT group (*p* = 0.0085, Tukey’s test) and to DHQ-treated FUS[1-359]-Tg mice (*p* = 0.0471, Tukey’s test; Figure 6B), making FUS[1-359]-Tg DHQ-treated group non-distinguishable from the untreated WT mice (*p* = 0.6964, Tukey’s test, Figure 6B).

### 3.6. Effects of DHQ Administration on Functional Outcomes and ALS Symptoms in FUS[1-359]-Tg and WT Littermates

No significant differences in the percentage of mice with ALS symptoms at the end of the experiment were observed between the DHQ-treated and untreated FUS[1-359]-Tg groups (*p* = 0.3405, Fisher’s exact test; Figure 6C). One-way ANOVA revealed significant group differences in the latency to descend in the pole test (F = 17.45, *p* < 0.0001). This measure was significantly decreased in both untreated and DHQ-treated FUS mice compared to the WT group (*p* < 0.0001 and *p* = 0.0004, respectively, Tukey’s test; Figure 6D). Significant group differences were observed in the latency to fall in the wire test (F = 8.498, *p* = 0.0009, one-way ANOVA). This indicator was significantly reduced in both the untreated and DHQ-treated FUS groups compared to that in the WT mice (*p* = 0.0063 and *p* = 0.0014, respectively; Tukey’s test; Figure 6E). Latency to fall from the rotarod also differed significantly between the groups (F = 8.642, *p* = 0.0008, one-way ANOVA). Similarly, it was significantly shortened in both the untreated and DHQ-treated FUS groups compared to that in WT mice (*p* = 0.0003 and *p* = 0.0167, respectively, Tukey’s test; Figure 6F).

One-way ANOVA revealed significant group differences in body weight changes (F = 10.77, *p* = 0.0002). This measure was significantly lower in untreated FUS[1-359]-Tg mice than in both the untreated WT group and DHQ-treated FUS[1-359]-Tg group (*p* = 0.0001 and *p* = 0.0482, respectively, Tukey’s test; Figure 6G). Liquid intake also differed significantly between the groups (F = 3.455, *p* = 0.0421, one-way ANOVA). This indicator was significantly lower in untreated FUS[1-359]-Tg mice than in the DHQ-treated FUS[1-359]-Tg group (*p* = 0.0406, Tukey’s test; Figure 6H). Significant group differences were found in dietary intake (F = 25.17, *p* < 0.0001, one-way ANOVA). It was significantly decreased in both untreated and DHQ-treated FUS[1-359]-Tg mutants compared to the untreated WT group (*p* < 0.0001 and *p* = 0.0004, respectively, Tukey’s test). At the same time, it was significantly higher in DHQ-treated FUS[1-359]-Tg mice than in the untreated FUS[1-359]-Tg group (*p* = 0.0146; Figure 6I). Finally, significant group differences were revealed for the coat state index (F = 18.45, *p* < 0.0001, one-way ANOVA). This measure was significantly lower in both untreated and DHQ-treated FUS[1-359]-Tg mutants than in the untreated WT group (*p* < 0.0001 and *p* = 0.0013, respectively, Tukey’s test; Figure 6J) with a strong trend for it to be elevated in DHQ-treated FUS[1-359]-Tg mutants than in the untreated FUS[1-359]-Tg group (*p* = 0.0707). However, the percentage of mice with a poor coat score of <2.5 was significantly higher in vehicle-treated mutants than in DHQ-treated FUS[1-359]-Tg mice (*p* = 0.0481, Fisher’s exact test; Figure 6J).

## 4. Discussion

The present study investigated whether chronic administration of a low dose of DHQ dissolved in water partially modifies disease-relevant pathological outcomes in two genetic models of neurodegeneration: AD and ALS. Across both models, we found that treatment with DHQ partially protected against the histological hallmarks of these disorders. In APPswe/PS1dE9 mice, DHQ reduced the density of small amyloid plaques in the cortex and thalamus and improved associative and spatial learning and anxiety-like behavior. These effects in DHQ-treated mutants might be due to a decrease in the oxidative stress marker, MDA, in the prefrontal cortex. Contextual learning and hippocampus-dependent performance in the pellet displacement test were not ameliorated by DHQ administration, consistent with the lack of significant improvement in the hippocampal density of amyloid plaques and unchanged astrocyte activation in the brain, suggesting partial translation of neuroprotective effects of the treatment to functional outcomes. In FUS[1-359]-Tg mice, an established model of ALS, DHQ administration increased the number of preserved ventral horn motor neurons and improved the general physiological parameters of mutants, such as water and diet intake, body weight, and coat state. However, changes in the onset of paralysis did not reach statistical significance, and motor performance in a battery of tests was not significantly altered by the treatment. Thus, chronic treatment with low doses of water solutions of DHQ ameliorated the histological hallmarks of both AD and ALS in the employed genetic models of these disorders, which were partially translated to functional improvements. Generally, this suggests the potential of using an aqueous solution of DHQ, a widely accessible compound extracted from numerous plants around the globe, for the prevention and treatment of genetically driven neurodegeneration.

Over the past decades, numerous efforts have been made to develop DHQ derivatives with improved aqueous solubility, bioavailability, and therapeutic efficacy, particularly for central nervous system disorders [7,11]. However, the use of these often-expensive formulations remains challenging, as the delayed appearance of DHQ in the brain suggests that transport across the BBB, rather than solubility alone, may be the principal limiting factor [10,15,24,78]. In addition, although the safety profile of native DHQ has been extensively established, it has not been established for its modified formulations [7,8,16]. Importantly, DHQ is an inexpensive, widely available natural compound that can be readily implemented in resource-limited settings, making the demonstration of its efficacy following administration as a simple aqueous solution of considerable translational and societal interest [1,11]. Furthermore, whereas the vast majority of previous studies have examined DHQ in in vitro systems [5,6,14,79] or toxicity-induced models of neurodegeneration [12,13,19], data on genetic models of disease are scarce. The present work showed the beneficial effects of continuous oral administration of DHQ in two well-characterized genetic models with high construct validity and established pharmacological sensitivity recapitulating pronounced neuropathology, such as aged APPswe/PS1dE9 mice and symptomatic FUS[1-359]-Tg mice.

The current results obtained in the genetic AD model are consistent with previous studies showing that by 12 months of age, APPswe/PS1dE9 mice are characterized by amyloid deposition and cognitive and emotional abnormalities [31,32,33,80]. DHQ treatment reduced small plaques (≤100 μm^2^) in the cortex and thalamus, whereas larger plaques were not significantly altered. In the hippocampal formation, these changes approached a level of statistical trend but were not significant. Our previous studies have demonstrated the physiological importance of amyloid plaque size, as the density and size of plaques correlate with elevated anxiety-like behavior and behavioral invigoration, as well as abnormal expression of molecular markers of AD at early stages of pathology in APPswe/PS1dE9 mice [76]. These markers included TNF, Il-6, SYP, IGF1, MAOB, and others, whose functions are related to inflammation, metabolic regulation, and plasticity, and are established targets of DHQ [7,8].

Of interest, similarly specific effects on small plaques (≤100 μm^2^) of antioxidant and anti-inflammatory interventions with fullerene compounds, agglutinin-based extract and stem cell therapy were shown in recent studies with APPswe/PS1dE9 mouse line [31,32,33,34]. This size- and region-selective effect suggests that DHQ administration may preferentially interfere with early plaque nucleation, small aggregate accumulation, or ongoing β-assembly rather than clearing already established amyloid deposits throughout the brain. This is also keeping with previous findings demonstrating the beneficial effects of DHQ on amyloidosis, mediated via counteraction of amyloid oligomerization [9,48,79], a key process of amyloid plaque formation and a key mechanism of neurodegeneration in AD [81,82].

This interpretation agrees with that of prior study on the use of DHQ in Aβ-related pathology in vitro [79] and in vivo [9,48,78]. For example, in Tg-SwDI mice model of cerebral angioamyloidosis, prolonged oral dosing with DHQ inhibited β-oligomer formation, reduced vascular Aβ accumulation, promoted Aβ clearance, restored cerebrovascular reactivity, and normalized spatial reference memory [9,48]. These effects were associated with ameliorated TLR4/NF-κB/NLRP3/caspase-1 signaling, downregulation of TREM2, activation of the Nrf2/HO-1 and SIRT-1-mediated pathways, as well as decreases in MDA and catalase levels, and suppressed expression of iNOS, COX-2, TNF, and IL-1 β [9]. These and other observations, apart from interference with Aβ aggregation, suggest the antioxidant and anti-inflammatory activity of DHQ, as shown in our study, as well as metal chelation, anti-glycation, and anti-inflammatory actions [78,83,84]. The present data extend this literature by showing that a water solution of DHQ can produce partial though measurable anti-amyloid, antioxidant, and behavioral effects in genetic amyloidosis model.

DHQ administration to APPswe/PS1dE9 mice improved conditioned taste aversion and Y-maze performance, including the number of correct choices, and decreased anxiety-like behavior in the elevated O-maze. These results indicate the beneficial effects of a low dose of DHQ on the form of memory that is impaired in AD patients in the first place. Improvement in learning can be underpinned by significant small plaque reduction in the cortex and thalamus of aged APPswe/PS1dE9 animals, as memory acquisition in both the conditioned taste aversion and spatial version of the Y-maze is dependent on the intact structure and functions of these brain areas [85,86]. The absence of a significant effect of treatment in the step-down avoidance test and pellet displacement paradigm in mutants may be due to a lack of significant amelioration of amyloid plaque density in the hippocampus, as rodent performance in both models is considered primarily dependent on the intact functions of this brain structure [87,88]. In addition, unchanged astrogliosis scores in DHQ-treated mutants may be interpreted as a sign of anti-inflammatory effects that are insufficient to rescue normal hippocampus-dependent performance, as the hippocampus is particularly sensitive to systemic inflammation [89,90]. The lack of a GFAP response suggests that the treatment used in the present study did not attenuate astrocyte activation in APPswe/PS1dE9 animals and that the cognitive benefit was mediated primarily through Aβ aggregation/clearance, oxidative stress, vascular function, or other mechanisms that were suggested for DHQ, for example, its effects on systemic inflammation and involvement of peripheral organs in these processes, as well as the effects of DHQ on the microbiome [48,91,92,93,94].

Here, tests for emotionality showed that DHQ-induced improvement in anxiety scores in the elevated O-maze and dark–light box, where such manifestations of anxiety-like behavior, such as reduced number of exits to anxiogenic areas and time spent therein, were reduced in vehicle-treated mutants. APPswe/PS1dE9 mice that received DHQ did not exhibit such abnormalities. In addition, the latency to exit to the lit compartment was normalized in the dosed versus vehicle-treated mutants, further suggesting the anti-anxiety effects of chronic dosing with a low dose of DHQ. These effects are likely mediated by the amelioration of plaque formation in the thalamus, as plaque accumulation within this structure is associated with anxiety-like behaviors [95]. The thalamus is increasingly recognized as a site of AD pathology [96], and its role in sensory integration and emotional regulation suggests its possible key role in the anti-anxiety effects of DHQ, the dosing of which significantly decreased small plaque density [97,98].

Given that the so-called ‘behavioral symptoms’ of AD, such as high anxiety, aggression, agitation, and panic behavior, are considered serious symptoms of this disorder as a cognitive disability [99,100,101], the findings regarding the positive effects of a low dose of DHQ on these abnormalities can be regarded as very valuable. The importance of amyloid plaque formation in the thalamus for the manifestation of anxiety-like behavior in the dark–light box was recently shown in APPswe/PS1dE9 mice [76]. In addition, a reduction in oxidative stress, as shown by a decrease in MDA levels in DHQ-treated mice, can underlie the normalized parameters of anxiety-like behavior, as suggested by the accumulated literature linking oxidative stress and AD pathology in clinical and pre-clinical studies [102].

The present study did not reveal any significant changes in the open field parameters of behavior, which is in accordance with our previous observations obtained in 12-month-old APPswe/PS1dE9 mice [31,32,33,34]. A lack of these changes rules out possible confounds in the evaluation of the above-described measures of emotionality and cognition and suggests the specificity of the revealed behavioral changes resulting from genetic mutation, the use of the DHQ, and their interactions. In addition, these data suggest that DHQ did not induce any signs of toxicity in aged control and APPswe/PS1dE9 mutants despite prolonged administration.

The present findings in FUS[1-359]-Tg mice, a model that recapitulates key features of FUS-associated ALS [35,103,104] showed that 6-week dosing with a low dose of DHQ rescued the number of Nissl-positive lower motor neurons and preserved normal motor neuronal density. This suggests that chronic administration of a low dose of DHQ can reduce motor neuron degeneration in a fast-progressing ALS model, the FUS[1-359]-Tg mice. Our data are consistent with recent observations showing that DHQ can prevent spinal motor neuron degeneration induced by chronic excitotoxic stimuli, which implicates a sirtuin 1-dependent mechanism [105].

The neuroprotective effects of DHQ on spinal motor neurons observed in the present study may be mediated by neuroinflammatory and oxidative stress pathways implicated in FUS-associated ALS. Mutant FUS disrupts NEAT1/paraspeckle function and promotes NLRP3 inflammasome activation, leading to IL-1β- and TNF-mediated motor neuron injury [45,46,47,106]. These pathways have been shown to be attenuated by DHQ through inhibition of TLR4/NF-κB/NLRP3/caspase-1 signaling, downregulation of TREM2, and activation of the Nrf2/HO-1 pathway [9,50,51,52]. Given the multifactorial nature and rapid progression of FUS proteinopathy, the observed neuroprotective effects are likely insufficient to significantly slow disease progression. This is consistent with the current view that anti-neuroinflammatory therapies alone exert modest disease-modifying effects in patients with ALS [53,107,108].

The neuroprotective effects reported here were accompanied by an improved general physical state, as shown by the physiological parameters of drinking behavior, body weight, and the percentage of mice with poor coat scores. The optical trend towards a later onset of ALS-like syndrome may explain the improved physiological parameters in DHQ-treated FUS[1-359]-Tg mice. However, key measures of disease progression, such as the age of onset of ALS-like symptoms and motor deficits, did not significantly improve with treatment. It can be suggested that the preservation of a subset of motor neurons may be insufficient to alter whole-animal motor performance once neuromuscular, glial, vascular, or muscle pathologies develop [109]. To further evaluate the therapeutic potential of DHQ in ALS and align with international recommendations for preclinical ALS research [57], it is important to validate our findings in an additional ALS model, such as SOD1 transgenic mice.

Indeed, the FUS[1-359]-Tg model progresses rapidly compared to other models of this disease, in which many powerful antioxidants effective in other models are ineffective [36,37,54]. Importantly, commonly accepted ALS therapies, such as riluzole and edaravone, exert only partial effects on motor function and the onset of ALS syndrome, as shown in clinical and pre-clinical studies. Our previous findings with riluzole, administered at a dose of 8 mg/kg/day to FUS[1-359]-Tg mice for 6 weeks, revealed a lack of significant effect on the onset of ALS pathology and only partially normalized the behavior of these mice in the wire test, pole test, and other paradigms for motor functions [53]. Thus, the present results with DHQ align with the majority of observations showing that most ALS therapeutics elicit only modest effects on this devastating pathology [36,37,110,111,112,113].

## 5. Conclusions

Taken together, our findings suggest that continuous oral administration of a low-dose aqueous solution of DHQ can partially counteract genetically driven neurodegeneration in two distinct disease models, although these histological benefits did not fully translate into functional and histological improvements. The dosing regimen was designed to account for the rapid gastrointestinal absorption, short systemic half-life, lack of substantial tissue accumulation, and the observation that DHQ BBB penetration is not markedly enhanced by higher doses or modified formulations [7,8,17,24]. Furthermore, the use of regular water as the vehicle, selected based on the established DHQ stability at neutral pH and room temperature, may provide a simple, safe, and inexpensive mode of medicinal use for this widely available natural compound, including in economically disadvantaged societies [114].

Our study is the first to demonstrate the biological effects of a water solution of DHQ used at low concentrations, but it has several limitations. First, further studies are required to perform dose–response assay, BBB permeability, and pharmacokinetic analysis. Second, soluble Aβ species and synaptic proteins were not measured directly, and this remains to be confirmed in future studies. Third, the group sizes of the APP/PS1 transgenic groups were small, allowing the design to resolve only large treatment effects, thus requiring their replication in larger cohorts of mice. In addition, larger group sizes and the use of alternative genetic models of AD and ALS conducted in line with the key features of the models [57,115,116,117] can help further investigate the neuroprotective properties of DHQ as this compound produced only partial histological and functional effects in our study.

## Data Availability

The original contributions presented in this study are included in the article/Supplementary Materials. Further inquiries can be directed to the corresponding author.

## Supplementary Materials

The following supporting information can be downloaded at: https://www.mdpi.com/article/10.3390/cells15171598/s1.

## Abbreviations

The following abbreviations are used in this manuscript:

AD	Alzheimer’s disease
ALS	Amyotrophic lateral sclerosis
Aβ	Amyloid β
APP/PS1	APPswe/PS1dE9 mice
CuZn-SOD	Copper–zinc superoxide dismutase
DHQ	Dihydroquercetin
FUS	Fused in sarcoma
GFAP	Glial Fibrillary Acidic Protein
Il1β	Interleukin 1 beta
Il6	Interleukin 6
LiCl	Lithium chloride
MDA	Malondialdehyde
Mn-SOD	Manganese superoxide dismutase
NaCl	Sodium chloride
NAD+	Nicotinamide adenine dinucleotide
NF-κB	Nuclear factor kappa B
PBS	Phosphate-buffered saline
PD	Parkinson’s disease
ROS	Reactive oxygen species
SEM	Standard error of measurement
Sirt1	Sirtuin 1
TLR4	Toll-like receptor 4
TNF	Tumor Necrosis Factor
WT	Wild-type
