# Supplementary material for: Efficacy of Low Dose of Dihydroquercetin (DHQ) in Two Genetic Models of Neurodegeneration: Insights from FUS[1-359]-Tg and APPswe/PS1dE9 Paradigms
Source: Cells. 2026 Sep 2;15(17):1598. doi: 10.3390/cells15171598 (PMC13565300; doi:10.3390/cells15171598)
Supplement: Supplementary file 1 [file cells-15-01598-s001.zip › cells-4514885-supplementary.pdf]

Supplementary File

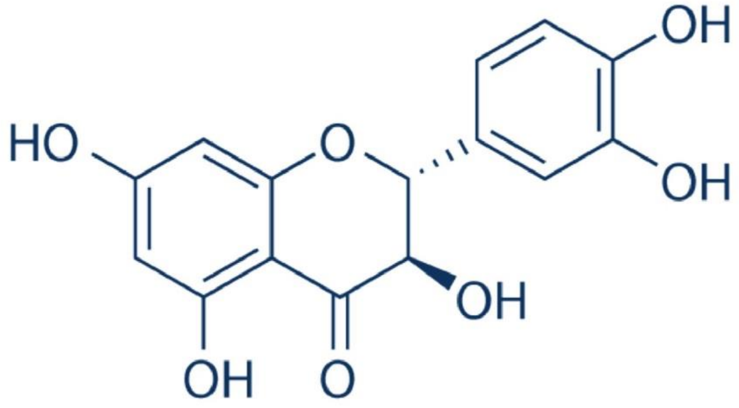

Supplementary Figure S1. Chemical structure of DHQ (**Liu et al., 2022).**

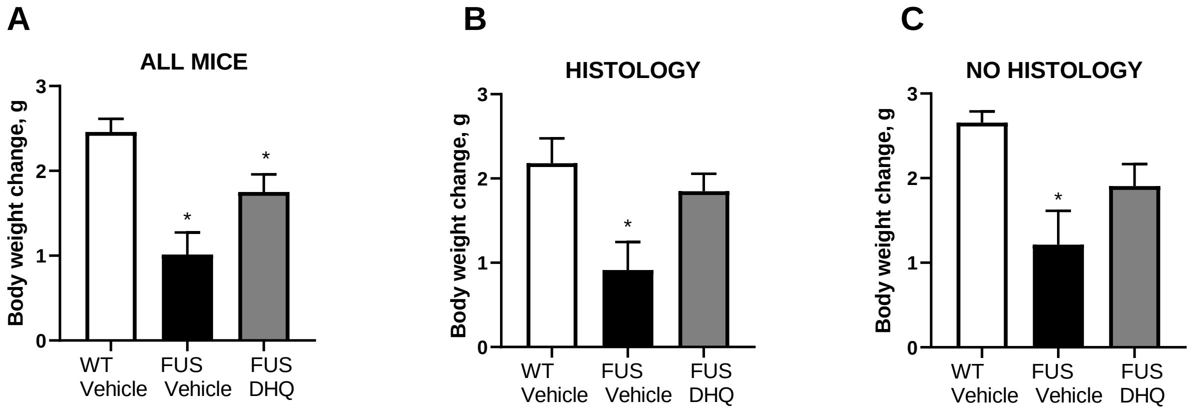

Supplementary Figure S2. Groups of mice from a study on FUS[1-359]-tg mice used for histological assay and spared for biochemical assay were balanced based on their body weight changes. (A) A comparison of all mice used in the study showed that both FUS-Tg mice treated with vehicle (n=14) and FUS-Tg mice treated with DHQ (n=14) displayed a significant reduction in weight gain compared to WT vehicle-treated mice (n=12). **(B,C)** Mice assigned for histological studies and additional biochemical assays displayed similar patterns of group differences in body weight changes. In both cohorts of animals, FUS-Tg mice treated with the vehicle (n=7 in each cohort) and FUS-Tg mice treated with DHQ (n=7 in each cohort) in each cohort) displayed reduced weight gain compared to WT-vehicle-treated mice (n=6 in each cohort), which reached a significant level for vehicle-treated mutants. *$p<0.05$, one-way ANOVA. Data are SEM±Mean.

Supplementary Table S1

Group of mice	Day1	Day2	Day3	W1	W2	W4	W8	W12	W16
WT-Veh	2.77±0.32	2.83±0.20	2.69±0.41	2.75±0.67	2.87±0.20	3.05±0.36	2.98±0.47	3.35±0.29	3.51.33
WT-DHQ	2.20±0.79	2.83±0.57	2.95±0.27	2.77±0.28	2.89±0.88	3.15±0.59	3.03±0.25	3.44±0.19	3.62±0.49
APP/PS1 Veh	2.69±0.21	2.75±0.24	2.25±0.18	2.65±0.39	3.11±0.60	2.90±0.18	3.09±0.23	3.66±0.20	3.34±0.28
APP/PS1 DHQ	2.35±0.85	2.88±0.32	3.04±0.51	3.01±0.49	2.87±0.43	3.04±0.36	3.02±0.24	3.11±0.39	3.45±0.25

Supplementary Table S1. Liquide intake in a study with APPswe/PS1E9 mice. There were no significant group differences between the groups at selected time points of DHQ dosing at the beginning of drug administration (day 3) as well as at the end of a 4-months (16-weeks) dosing. Data are shown in mL as Mean ± SEM.

Supplementary Table S2. Liquide intake in a study with FUS[1-359]-tg mice

Group of mice	Day1	Day2	Day3	W1	W2	W3	W4	W5	W6
WT-Veh	2.67±0.22	2.83±0.20	2.69±0.41	2.75±0.67	2.97±0.22	3.10±0.16	2.98±0.18	3.15±0.15	3.11.13
FUS-tg Veh	2.56±0.27	2.80±0.23	2.75±0.28	2.89±0.39	2.91±0.32	2.870±0.19	1.92±0.25	1.55±0.20	1.64±0.29
FUS-tg DHQ	2.68±0.35	2.06±0.52	2.94±0.61	3.01±0.29	2.87±0.23	3.04±0.36	2.39±0.21	2.36±0.25	2.42±0.35

Supplementary Table S2. Liquide intake in a study with FUS[1-359]-tg mice. There were no significant group differences between the groups at selected time points of DHQ dosing at the beginning of drug administration (day 3) as well as at the end of a 6- week dosing. Data are shown in mL as Mean ± SEM.
